# Neurofind: using deep learning to make individualised inferences in brain-based disorders

**DOI:** 10.1038/s41398-025-03290-x

**Published:** 2025-02-27

**Authors:** S. Vieira, L. Baecker, W. H. L. Pinaya, R. Garcia-Dias, C. Scarpazza, V. Calhoun, A. Mechelli

**Affiliations:** 1 Department of Psychosis Studies, Institute of Psychiatry, Psychology & Neuroscience, King’s College London, London, UK; 2 Department of Radiology, Lausanne University Hospital and University of Lausanne (CHUV-UNIL), Lausanne, Switzerland; 3 Center for Research in Neuropsychology and Cognitive Behavioural Intervention, Faculty of Psychology and Educational Sciences, University of Coimbra, Coimbra, Portugal; 4 Department of Biomedical Engineering, King’s College London, London, UK; 5 Department of General Psychology, University of Padova, Padova, Italy; 6 IRCCS S Camillo Hospital, Venezia, Italy; 7 Tri-Institutional Center for Translational Research in Neuroimaging and Data Science (TReNDS) [Georgia State University, Georgia Institute of Technology, and Emory University], Atlanta, GA USA

**Keywords:** Neuroscience, Biomarkers

## Abstract

Within precision psychiatry, there is a growing interest in normative models given their ability to parse heterogeneity. While they are intuitive and informative, the technical expertise and resources required to develop normative models may not be accessible to most researchers. Here we present Neurofind, a new freely available tool that bridges this gap by wrapping sound and previously tested methods on data harmonisation and advanced normative models into a web-based platform that requires minimal input from the user. We explain how Neurofind was developed, how to use the Neurofind website in four simple steps (www.neurofind.ai), and provide exemplar applications. Neurofind takes as input structural MRI images and outputs two main metrics derived from independent normative models: (1) Outlier Index Score, a deviation score from the normative brain morphology, and (2) Brain Age, the predicted age based on an individual’s brain morphometry. The tool was trained on 3362 images of healthy controls aged 20–80 from publicly available datasets. The volume of 101 cortical and subcortical regions was extracted and modelled with an adversarial autoencoder for the Outlier index model and a support vector regression for the Brain age model. To illustrate potential applications, we applied Neurofind to 364 images from three independent datasets of patients diagnosed with Alzheimer’s disease and schizophrenia. In Alzheimer’s disease, 55.2% of patients had very extreme Outlier Index Scores, mostly driven by larger deviations in temporal-limbic structures and ventricles. Patients were also homogeneous in how they deviated from the norm. Conversely, only 30.1% of schizophrenia patients were extreme outliers, due to deviations in the hippocampus and pallidum, and patients tended to be more heterogeneous than controls. Both groups showed signs of accelerated brain ageing.

## Introduction

Psychiatric and neurological disorders represent 10.4% of the global burden of disease and account for 258 million DALYs per year [1]. Until recently, the investigation of the brain correlates of these disorders has relied mostly on analytical methods based on mass-univariate comparisons between groups (e.g., statistical parametric mapping) [2–4]. However, this approach is not aligned with the complex, widespread and heterogeneous alterations which are typical of these disorders and the need for individual-level decisions in clinical practice [2, 5, 6]. This discrepancy may explain the limited translational impact of neuroimaging findings in neurology and psychiatry so far [7]. In recent years, a growing number of studies have attempted to address this issue by using multivariate predictive methods that allow statistical inferences at the individual level [8–11]. Within this movement, there is a growing interest in normative models given their added ability to parse heterogeneity [12–14]. Here, the aim is to first model the variability of a measure (e.g., brain morphology) or relationship (e.g., brain morphology and age) of interest in a reference cohort. Because the latter is typically a group of healthy controls (HC), it is assumed that the variability captured by the model corresponds to the ‘norm’, i.e., expected pattern in the absence of illness. A new and unseen individual can then be mapped against this model, and the distance of this individual from the norm can be quantified. Within this approach, individuals with the same diagnosis can differ from healthy controls in their own unique way or, importantly, not differ at all (i.e., not deviate from the norm) [15]. This is a significant departure from the traditional case-control design, where the ‘average patient’ is compared with the ‘average control’, under the assumption that they represent qualitatively different groups. This may be a reasonable assumption when the patient group is characterised by consistent and well-defined alterations. However, a growing number of studies using normative modelling are revealing that a surprisingly large proportion of patients fall within the normative range for several brain features [16, 17] and that there is substantial heterogeneity within diagnoses [6, 16, 18–22] as well as overlap between diagnoses [18].

Following these advances and potential for clinical translation, several web-based tools have been developed. However, as we discuss in a recent systematic review [23], these tools were designed to support the diagnosis of neurological disorders namely dementia (ADABOOST [24], Jung Diagnostistics [25, 26], NeuroQuant [27], Quantib [28], volBrain [29, 30]), multiple sclerosis (Jung Diagnostics [31], Quantib [32], volBrain [33]), traumatic brain injury (Icobrain [34], NeuroQuant [35–38]), temporal lobe epilepsy (NeuroQuant [39, 40]) and intracranial hemorrhages and mass effects in the brain (Qure [41]). These tools map known biomarkers for these disorders including atrophy of hippocampal/sub-cortical structures and ventricles (NeuroQuant, Jung Diagnostics), white-matter lesions (Icobrain, Jung Diagnostics, Quantib, volBrain) or gross abnormalities with computed tomography (Qure). Therefore, to our knowledge, there are no easy-access tools to map deviations from normative whole-brain morphology without making assumptions about a possible diagnosis of a neurological disorder. This hinders the use of existing tools in psychiatry, where disorders are characterised by a heterogeneous, subtle and widespread pattern of abnormalities across the brain. Here we present *Neurofind*, a new user-friendly and freely available web-based tool that outputs an individualised report based on normative modelling from high-resolution structural magnetic resonance (MRI) images. This individualised report includes two main components: (1) Outlier Detection - overall deviation from the normative brain morphology as well as deviations for each brain region, and (2) Brain Age - predicted age based on an individual’s brain morphology. The Outlier Detection model uses an adversarial auto-encoder (AAE), a deep learning approach that has shown promising results in clinical neuroimaging [42, 43]. Specifically, we developed an AAE that learns a latent representation of the input data by first reducing its dimensionality via consecutive layers of non-linear transformations (encoder); this reduced representation is then used to reconstruct the input (decoder). By constraining this reconstruction to be as similar as possible to the input data, the autoencoder is forced to learn a good representation of the input data [44]. Therefore, the AAE will be able to reconstruct new data with minimal difference between input and reconstruction if it is similar to the data used to learn the latent representation. In Neurofind, we used an AAE to learn a latent representation of the normative brain morphology. It is expected that, when presented with new images, the AAE will be able to reconstruct data from healthy controls, but it will be less precise when processing data from patients, i.e., the reconstruction error (i.e., difference between input and reconstruction) will be larger in patients compared to healthy controls. Therefore, the reconstruction error can be thought of as a proxy for deviation from the normative disease-free brain morphology. The Brain Age model, on the other hand, measures the effects of ageing on the brain and builds on the well-established relationship between age and neuroanatomy across the lifespan [45]. Brain age is often used to calculate the brain age gap (BAG), i.e., the difference between the predicted brain age and chronological age. A positive BAG means that the individual’s predicted brain age was higher than their actual age, suggesting ‘accelerated’ ageing; conversely, a negative BAG indicates ‘delayed’ ageing. There has been a surge in the investigation of BAG as a potential biomarker [46] after several studies showing an higher brain ages in psychosis [47–51], bipolar disorder [50–53], Alzheimer’s disease (AD) [51, 54, 55], among others. In addition to providing these two informative and complementary metrics, Neurofind uses a novel method – Neuroharmony [56] – to mitigate scanner effects of new incoming images. This overcomes the limitation of popular harmonisation methods, such as ComBat [57, 58], that require a representative sample from each scanner, which is not suitable for clinical translation, as new patients’ scans will likely originate from previously unseen scanners. Therefore, Neurofind combines our previous work on the use of AAE to build a normative model of brain morphology [59], brain age [60] and harmonisation of unseen scanners [56] into a user-friendly web-based tool targeted at researchers who wish to apply normative modelling to their morphological data. In the following sections, we present (1) the development of the models in Neurofind, (2) a guide on how to use Neurofind in four simple steps, and (3) exemplar applications of the tool in AD and schizophrenia (SZ).

## Model development

### Datasets

Data for training the web-based normative models used in Neurofind consisted of four publicly available datasets of healthy controls, namely the Human Connectome Project [61, 62], the Human Connectome Project Aging [63], Biobank [64] and IXI (http://brain-development.org/ixi-dataset/).

### MRI data acquisition and pre-processing

**Fig. 1:**
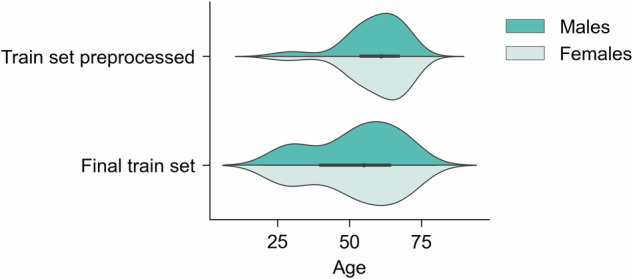
Distribution of age and sex in the train set before and after addressing data imbalance.

The initial total sample included 13,918 T1-weighted MRI images from 7 scanners (see Supplementary Materials for image acquisition parameters). Participants with missing data and younger than 20 and older than 80 years old were excluded. Poor quality images were excluded based on the MRIQC tool [65]. This tool uses 68 image quality metrics (IQMs) such as the presence of movement, artefacts, and signal-to-noise ratio to determine the probability of an image being unusable. Images with a probability higher than 0.7 were discarded. The remaining images were pre-processed using the recon-all pipeline with standard parameters in FreeSurfer (version 6.0) [66]. The cortical surface was parcellated using the Desikan-Killiany cortical atlas [67] and segmented into 68 cortical regions (34 per hemisphere). An additional 33 neuroanatomical structures were extracted using the ASEG atlas in FreeSurfer [66, 67] totalling 101 brain regions (ROIs) (full list of extracted regions in the Supplementary Materials). We estimated the relative brain region volumes (ROI_rel_) for each subject by dividing the original brain region volumes by the total intracranial volume (also computed with FreeSurfer). The final pre-processed data included 13,187 participants. However, there was an imbalanced distribution between the number of younger and older healthy controls, mostly due to the Biobank dataset (Fig. 1). The largest scanner of the Biobank dataset was under-sampled such that a maximum of 55 participants per age group were selected at random. Since outliers are unexpected in healthy subjects and are likely to be due to artefacts, healthy controls in the train set with at least 10 ROI_rel_ more than 3 standard deviations (σ) away from the sample mean (μ) were considered outliers and excluded. This process was repeated iteratively, recalculating μ and σ until no additional participant met the criteria for being an outlier. This process was implemented within each scanner to ensure that participants would not be considered outliers simply due to differences between scanners. The final data for the train set comprised 3362 participants (Fig. 1, Table 1, Supplementary Materials for sample size/scanner).

**Table 1:** Demographic characteristics of the train set used in model development.

	Train set (*N* = 3362)
Sex F/M, *N* (%)	1893 (56.3) / 1469 (43.7)
Age, median [Q1, Q3]	52.0 [15.2]

### Scanner harmonisation

Scanner effects on the ROI_rel_ were mitigated using the Neuroharmony method [56]. Briefly, Neuroharmony uses a random forest model to capture the relationship between IQMs and the corrections for each brain region prescribed by ComBat, a popular harmonisation approach that uses an empirical Bayes framework to correct for additive and multiplicative batch effects [68]. Once this relationship is learned, Neuroharmony predicts and applies the ComBat corrections for a new set of IQMs extracted from an unseen T1-weighted image. Neuroharmony was trained twice to accommodate the specific needs of the Outlier Detection and Brain Age models. For the former, Neuroharmony was trained whilst controlling for the effects of age and sex, whereas for Brain Age, only sex was added as a covariate. Neuroharmony was trained in the same train datasets as used for the development of the outlier detection and brain age models. Results of the harmonisation are shown in the Supplementary Materials.

### Outlier detection

Our outlier detection model uses an adversarial autoencoder (AAE) to learn a representation of disease-free brain morphology. Briefly, AAE combines a standard autoencoder with adversarial learning to improve the learned latent representation by forcing it to have a distribution similar to a desired prior distribution. In our approach, we used a Gaussian distribution. This is achieved by adding a third element to the standard autoencoder, the discriminator, responsible for deciding whether its input data comes from random numbers sampled from the predefined prior distribution or the latent code. A successful AAE will be able to generate a latent code capable of fooling the discriminator into inferring that the encoded samples come from the prior distribution (i.e., the discriminator will not be able to distinguish the two distributions). Therefore, the aim is to produce a latent representation that yields a low reconstruction error whilst also having a similar shape to the desired prior distribution. The AAE was trained in the harmonised ROI_rel_ from the train set following the same approach described in previous work [59] (see Supplementary Materials for details on model training). The reconstruction error for each ROI_rel_ was estimated by calculating the squared error between the inputted value and its reconstruction. An overall reconstruction error was then generated for each participant by calculating the mean squared error between the reconstruction and the inputted data. The distributions for all ROI-level and overall reconstruction errors in the train set were positively skewed. Therefore, all values were scaled by subtracting the median and dividing by the interquartile range such that the median of the overall and ROI-level scaled errors was 0.0 (IQR = 1.0) (Supplementary Materials). The final scaled errors, which we named Outlier Index Score (OIS), represent the number of interquartile ranges away from the median in the train set. The OIS is categorised into: Within the norm (<0.26), Low (≥0.26), Medium (≥1.1) and High (≥2.3) deviations from the norm (values indicate number of interquartile ranges away from the median in the train set). Cut-off values were extracted from the train set by first dividing the distribution of OIS into centiles and then grouping them into quartiles.

### Brain age

To predict brain age, harmonised and scaled ROI_rel_ from the train set were inputted to a linear support vector regression (SVR) model as implemented in the Python package scikit-learn [69] with a similar approach as described previously [60]. A systematic hyperparameter search for C was conducted using a ten-fold cross-validated grid-search over the search space 2^−7^, 2^−5^, 2^−3^, 2^−2^, 2^−1^, 2^0^, and 2^1^. The C value with the best performance, as determined by the scoring parameter negative mean absolute error (MAE), was then applied to the whole training set (C = 2^−2^). The parameters epsilon and tolerance for stopping criterion were 0.1 and 1e–3, respectively. In the train set, the MAE was 10.5 ± 9.8 years.

## How to use Neurofind

**Fig. 2:**
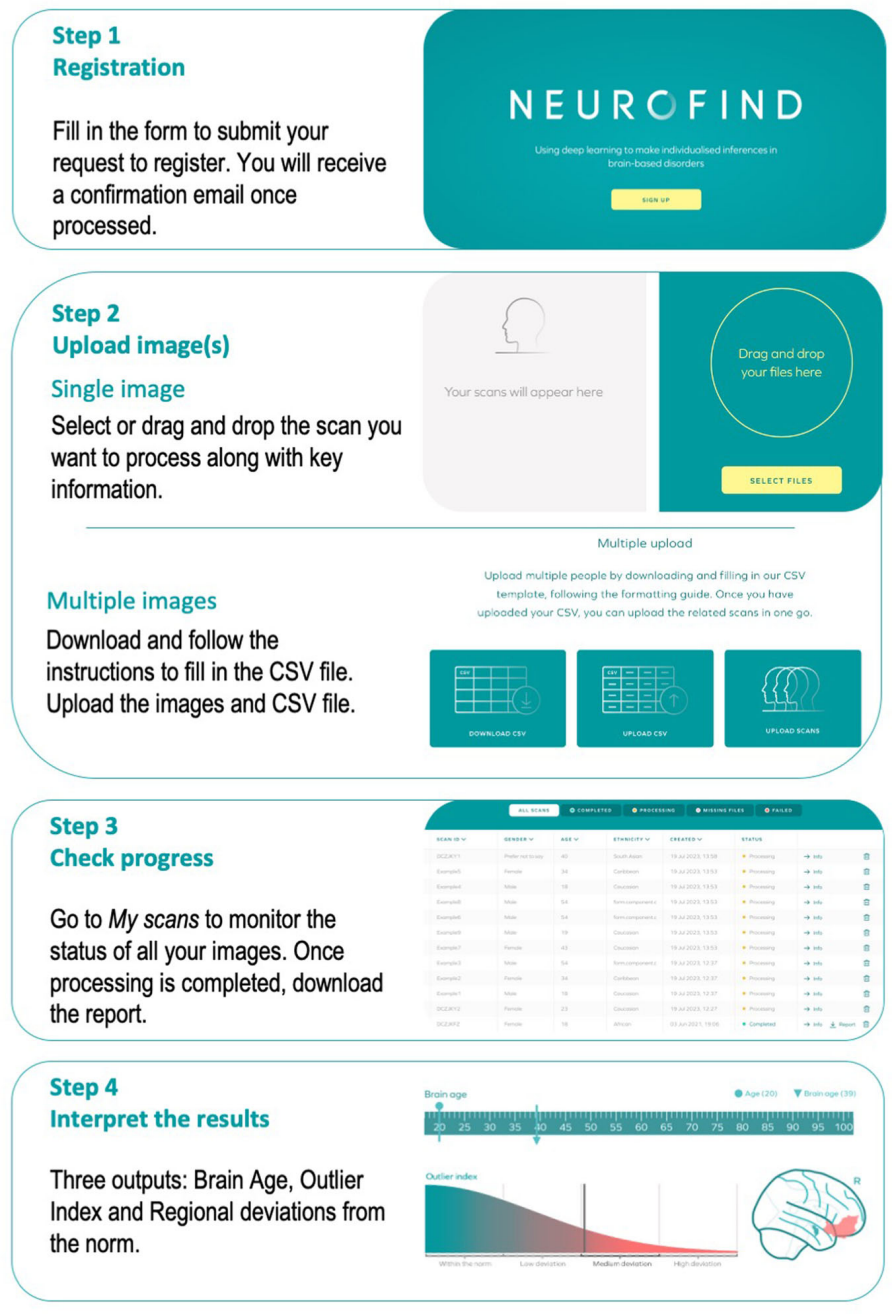
Main steps to use Neurofind.

Neurofind is a web-based research tool available at www.neurofind.ai. This section provides a step-by-step guide on how to get started, upload your images, check the status of your analyses as well as download and interpret the results. A graphical illustration of these steps can be found in Fig. 2. Details regarding how the data are stored and managed can be found in the Privacy Policy: https://neurofind.ai/privacy-policy.

### Step 1. Registration

To use Neurofind, you will first need to register a user account. You will be asked to provide simple key information (e.g., name, institution, role) and to read and accept the privacy policy. You will receive an email confirmation that your registration is approved.

### Step 2. Upload image(s)

Neurofind allows the processing of a single image or several images in bulk. To upload one image, choose Single upload from the top menu and fill in the necessary information. Upload your image by dragging it or clicking on Select files and navigating to the folder where the image is stored. Images can only be in NIfTI (.nii, .nii.gz) format. It is important that you have the subject’s consent to analyse their data in Neurofind, so the upload page also asks you to confirm that you have the necessary approvals before you can select Submit. To upload several images at once, select Multiple upload from the top menu. You will be asked to upload all your images at once along with a CSV file with all the relevant information about the images. To do this, select Download CSV to view the instructions on how to fill in the CSV file and download the template. It is important that you do not include any identifiable information in the CSV file or elsewhere on the website. Once completed, upload it by pressing Upload CSV. Next, click on Upload scans to upload the images. The processing of the image(s) will start as soon as the upload is completed.

### Step 3. Check progress

To monitor the progress of your analyses, go to My scans in the top menu. You will see the status of all images submitted for processing as well as ID, key demographic information, and time when processing began. The status of each image will be one of the following: Missing information, Processing, Completed or Failed. If any of the information requested in Step 2 is missing, processing will not begin automatically, and the status will be Missing information. To add missing information, go to My scans, find the image with the status Missing information and select Info. Once the missing information has been entered, processing will begin automatically, and the status will change to Processing. Processing includes the entire analysis, from image upload to results. This means that the pre-processing of the images (e.g., segmentation into different brain volumes) is also done automatically by Neurofind. If there is a problem during processing, the status will be changed to Failed. Possible reasons for this include poor-quality images or issues with network connection. If this occurs, you will need to reupload the image(s) from the beginning. When Processing is finished, the status will change to Completed and an individualised report will be available to view and download as a PDF file. You will also be notified via email once processing is complete.

### Step 4. Obtaining the results

The individualised report contains three main outputs: Brain Age, Outlier Index Score and Regional deviations from the norm. For Brain Age, the predicted and chronological ages are given. The difference between the two represents the BAG. A positive BAG means that the individual’s predicted brain age was higher than their chronological age, suggesting ‘accelerated’ ageing; conversely, a negative BAG indicates ‘delayed’ ageing. The Outlier Index Score conveys the magnitude of the overall deviation from the normative brain morphology. As the deviation increases, the Outlier Index Score will be classified as Within the norm, Low, Medium and High deviation from the norm (see Section ‘Outlier detection’ for explanation of cut-off values). The brain regions with the largest deviations from the norm are shown in a glass brain and shown in full in a table at the end of the report.

## Exemplar applications

### Datasets used for exemplar applications

Exemplar applications were carried out in three clinical datasets: the Australian Imaging, Biomarkers and Lifestyle Study (AIBL), the MIND Clinical Imaging Consortium (MCIC) and the Center for Biomedical Research Excellence (COBRE) dataset. The AIBL dataset includes patients diagnosed with AD and healthy controls; the latter two datasets include patients diagnosed with SZ and corresponding healthy controls; for details about recruitment see [70–73].

### MRI data acquisition and pre-processing

**Fig. 3:**
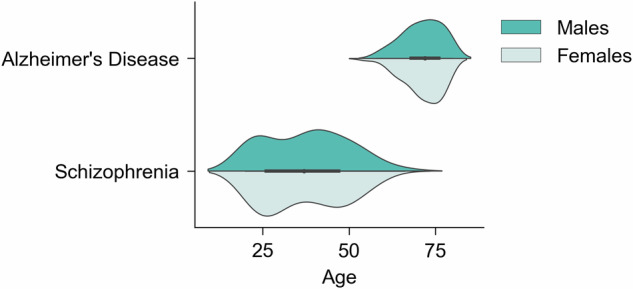
Distribution of age and sex in the clinical datasets used for exemplar applications.

The initial total sample for exemplar applications included 759 T1-weighted MRI images from 10 scanners (see Supplementary Materials for image acquisition parameters). Data were cleaned and pre-processed in line with the steps outlined for the model development data in Section ‘Model development’. In addition, healthy controls from AIBL were randomly subsampled to match the sample size of AD patients to address the large class imbalance. The final data comprised 364 participants in the clinical datasets (Fig. 3, Table 2, Supplementary Materials for sample size/scanner). Age did not follow a normal distribution in all sub-groups according to the Shapiro-Wilk test (AD_AD_ = 0.9, *p* < 0.001; AD_HC_ = 1.0, *p* < 0.05; SZ_SZ_ = 0.9, *p* < 0.001; SZ_HC_ = 0.9, *p* < 0.01). Differences in sex and age between patients and healthy controls in the clinical data were tested using chi-square test and Mann-Whitney U test, respectively; this revealed no statistically significant differences between the two groups (Table 2).

**Table 2:** Demographic characteristics of the clinical datasets used for exemplar applications.

	Clinical datasets (*N* = 367)					
	Alzheimer’s disease (*N* = 122)			Schizophrenia (*N* = 245)		
	AD (*N* = 61)	HC (*N* = 61)	*p*	SZ (*N* = 134)	HC (*N* = 111)	*p*
Sex F/M, *N* (%)	37 (60.7) / 24 (39.3)	43 (70.5)/ 18 (29.5)	0.341	30 (22.4) / 104 (77.6)	32 (28.8) / 79 (71.2)	0.314
Age, median [Q1,Q3]	73.0 [69.0,77.0]	71.0 [68.0,75.0]	0.244	35.0 [25.0,46.0]	37.0 [27.0,47.0]	0.282

### Scanner harmonisation

The Neuroharmony model (trained in the model development data, as described in Section ‘Model development’) was applied separately to the clinical datasets used for exemplar applications. Results of the harmonisation are shown in the Supplementary Materials.

### Statistical analyses in exemplar applications

The overall and ROI-level reconstruction errors in the clinical sample were scaled according to the median and interquartile range obtained in the train set (see Section ‘Outlier detection’). Differences in overall and ROI-level OIS and BAG between patients and respective controls were investigated using an independent-sample t-test or Mann-Whitney U Test depending on whether the distribution for both groups was normally distributed or not, respectively. Normality was tested using the Shapiro-Wilk test. For the ROI-level comparisons the p-value was adjusted using the Benjamini-Hochberg correction for multiple comparisons. The association between OIS and BAG with symptom severity was tested with Pearson’s correlation coefficient (r) or Spearman’s rho (*ρ*) depending on the outcome of the Shapiro-Wilk test for the symptoms. Statistical significance was set at 0.05. Patients’ symptom severity was assessed with the Mini Mental State Examination (MMSE) [74] (AIBL), the Scale for the Assessment of Negative Symptoms (SANS) [75] and the Scale for the Assessment of Positive Symptoms (SAPS) [76] (MCIC) or the Positive and Negative Syndrome Scale (PANSS) [77] (COBRE). Lower scores in the MMSE and higher scores in the psychosis scales indicate worse illness severity. The positive (PANSS Positive and SAPS) and negative (PANSS Negative and SANS) symptoms scales were normalised to ensure comparability across sites using the formula [4]:

$${\rm{New\; score}}=\frac{{\rm{Individual\; raw\; score}}-{\rm{Minimum}}}{{\rm{Maximum}}-{\rm{Minimum}}}$$

where Minimum and Maximum refer to the lowest and highest score allowed for either PANSS or SAPS/SANS. The resulting symptom severity scores were normalised by subtracting the mean from every item and then dividing the resulting value by the standard deviation of the item (i.e., zero mean unit variance normalization). To investigate heterogeneity of deviations from the normative brain morphology amongst participants, we calculated the mean pairwise cosine similarity (CS) across all individual ROI-level OIS and within predefined brain regions based on the Desikan-Killiany atlas: frontal, temporal, parietal and occipital lobes, insula, cingulate, subcortical structures, cerebellum, corpus callosum and ventricles (Supplementary Materials). Briefly, CS quantifies the similarity between two vectors by calculating the cosine of the angle between the two. The magnitude varies from 0 (low similarity between individuals, i.e., vectors are 90 degrees or perpendicular to each other) and 1 (perfect similarity between individuals). BAG was calculated by subtracting the chronological age from the predicted brain age and normalised according to the train set used for model development.

### Results from exemplar applications

#### Quantifying deviation from the normative brain morphology

**Fig. 4:**
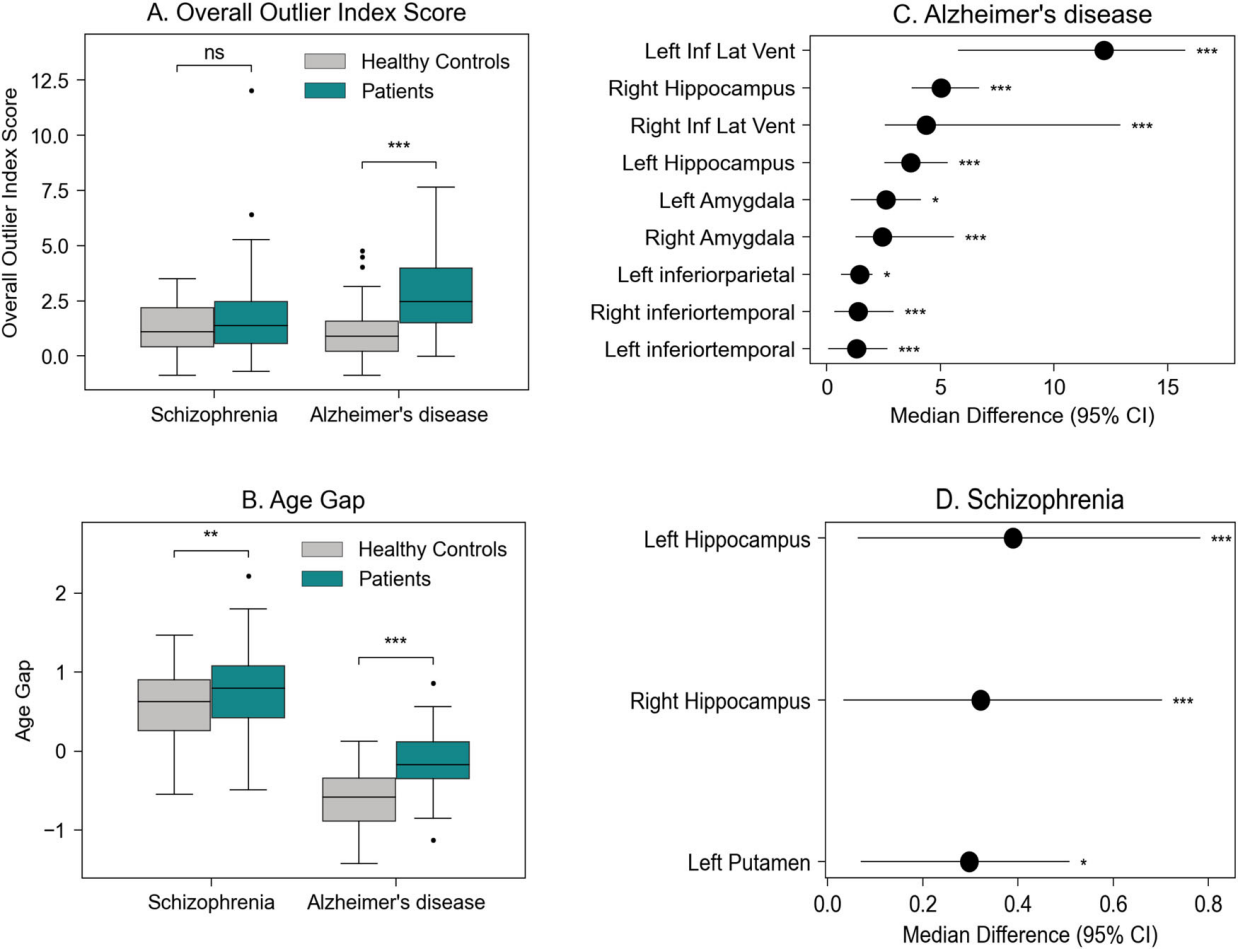
Results from the exemplar applications for AD and SZ. **A** Outlier Index Score (OIS) for AD and SZ. **B** BAG for patients and HC. **C**, **D** Median difference between OIS for schizophrenia / Alzheimer’s disease and respective controls for ROIs with a statistically significant difference between the two. Plots show the mean difference between patients and respective controls and the 95% confidence interval (CI) for this difference. The 95% CI was calculated with bootstrapping (1000 repetitions). ^***^p ≤ 0.001, ^**^p ≤ 0.010, ^*^p < 0.05, ns *p* > 0.05.

The overall and ROI-level OIS did not follow a normal distribution, whilst the BAG did (Supplementary Materials). AD patients (Median = 2.46, IQR = 2.50) showed a significantly larger OIS compared to HC (Median = 0.91, IQR = 1.36) (Mann-U = 1361; *p* < 0.001) (Fig. 4A). AD patients showed significantly larger median OIS for individual ROIs relative to respective HC for bilateral amygdala (Mann-U_Left Amyg_ = 1196, *p* < 0.01; Mann-U_Right Amyg_ = 1246, *p* < 0.001), hippocampus (Mann-U_Left Hipp_ = 1476, *p* < 0.001; Mann-U_Right Hipp_ = 1425, *p* < 0.001), inferior temporal gyrus (Mann-U_Left Inf Temp_ = 1245, *p* < 0.001; Mann-U_Right Inf Temp_ = 1343, *p* < 0.001) and inferior lateral ventricle (Mann-U_Left Inf. Lat. Ven._ = 1519, *p* < 0.001; Mann-U_Right Inf. Lat.Ven._ = 1382, *p* < 0.001), left inferior parietal gyrus (Mann-U = 1185, *p* < 0.05) (Fig. 4C; see Supplementary Materials for detailed results). Conversely, there was no statistically significant difference in overall OIS between SZ patients (Median = 1.38, IQR = 1.83) and respective HC (Median = 1.10, IQR = 1.76) (Mann-U = 7657; *p* = 0.091) (Fig. 4A). However, patients showed significantly larger median deviations from HC in bilateral hippocampus (Mann-U _Left Hipp_ = 8077, *p* < 0.01; Mann-U_Right Hip_ = 7384, *p* < 0.05) and left putamen (Mann-U = 7846, *p* < 0.05) (Fig. 4D, see Supplementary Materials for detailed results).

**Fig. 5:**
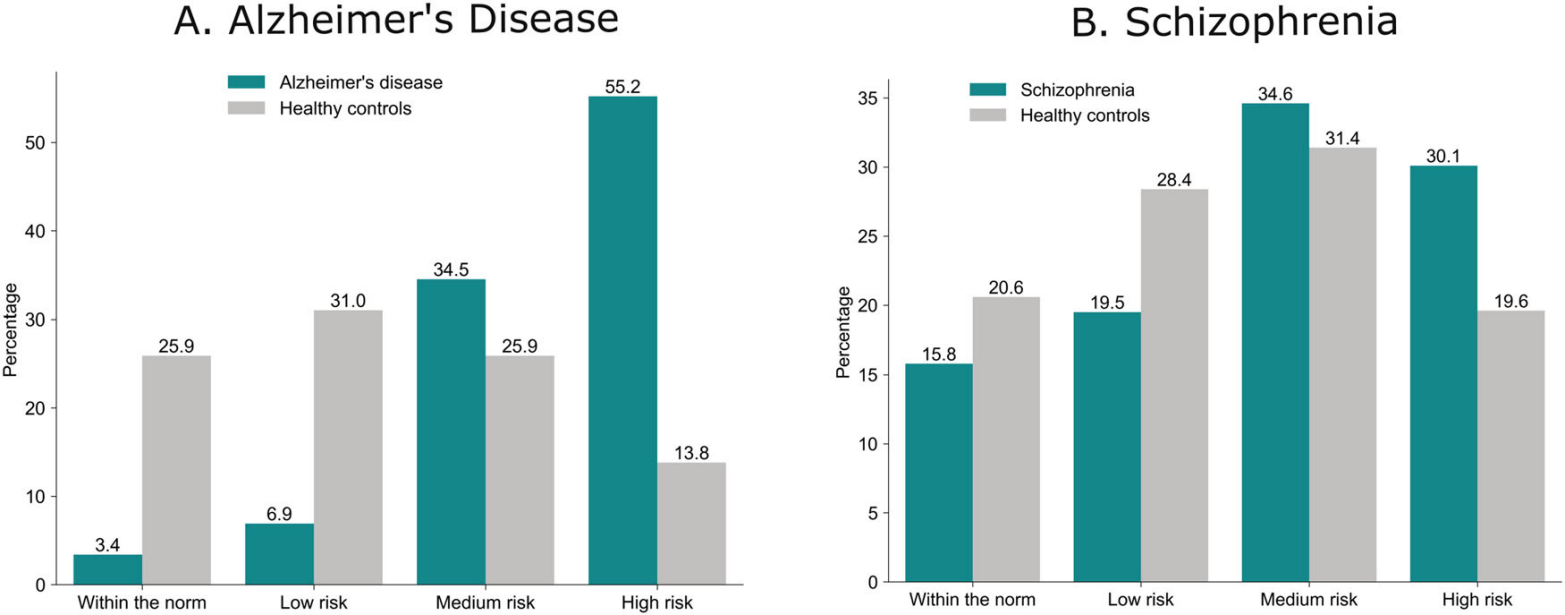
Proportion of patients with overall OIS within the norm, low, medium, or high deviation. **A** In the Alzheimer’s disease group, only a small fraction of patients exhibited an overall OIS within the normative range. The percentage of patients classified with deviations increased progressively, with the majority falling into the high deviation category. **B** In the schizophrenia group, a greater proportion of patients displayed deviations from the norm, with more individuals classified in the medium and high deviation categories than in the low deviation range.

Only 3.4% of AD patients against 25.9% HC had an overall OIS within the norm (Fig. 5). The proportion of AD patients with low, medium and high deviation relative to HC increased steadily such that 55.2% of patients and only 13.8% HC had high deviations from the norm. Conversely, 15.8% of SZ patients and 20.6% of HC had an overall normative brain morphology. However, there was higher proportion of SZ patients in the medium and high deviation categories compared to HC.

#### Estimating brain age gap

The overall performance of the brain age model in the healthy controls of the clinical datasets was MAE 10.0 ± 6.3 years. The mean scaled BAG was significantly larger for AD (M = −0.1, SD = 0.4) compared to their respective HC (M = −0.6, SD = 0.4) (*p* < 0.001) as well as for SZ (M = 0.8, SD = 0.5) compared to their respective HC (M = 0.6, SD = 0.4; *p* < 0.01). This indicates that for both illnesses, the predicted brain age was higher than their expected age based on the control groups, suggesting ‘accelerated’ ageing (Fig. 4D).

#### Investigating the neural correlates of symptom severity

**Fig. 6:**
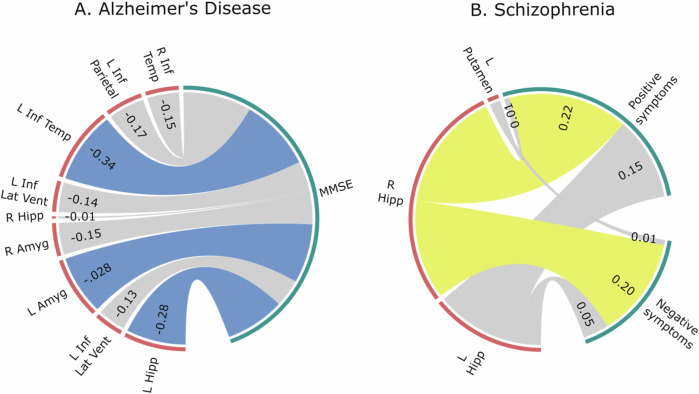
Spearman’s correlation between regions of interest (ROIs) with significantly larger deviations from the norm in patients and symptom severity. **A** In Alzheimer’s disease, greater deviations from the norm in the left hippocampus, left amygdala, and left inferior temporal gyrus were associated with increased disease severity, as indicated by significant negative correlations. **B** In schizophrenia, larger deviations in the right hippocampus were significantly associated with greater positive and negative symptom severity.

Severity of AD was negatively associated with the left hippocampus (*ρ* = −0.28, *p* < 0.05), left amygdala (*ρ* = −0.28, *p* < 0.05) and left inferior temporal gyrus (*ρ* = −0.34, *p* < 0.05). As expected, these negative associations indicate that the larger the deviation from the norm, the more severe the symptoms. In SZ, there was a significant positive association between right hippocampus and positive (*ρ* = 0.22, *p* < 0.05) and negative (*ρ* = 0.20, *p* < 0.05) symptoms (Fig. 6, see Supplementary Materials for detailed results). The association between BAG and symptom severity was statistically significant in SZ for positive (*ρ* = 0.20, *p* < 0.05) but not negative symptoms (*ρ* = 0.04, *p* = 0.680) nor MMSE in AD (*ρ* = −0.16, *p* = 0.266).

#### Estimating heterogeneity

**Fig. 7:**
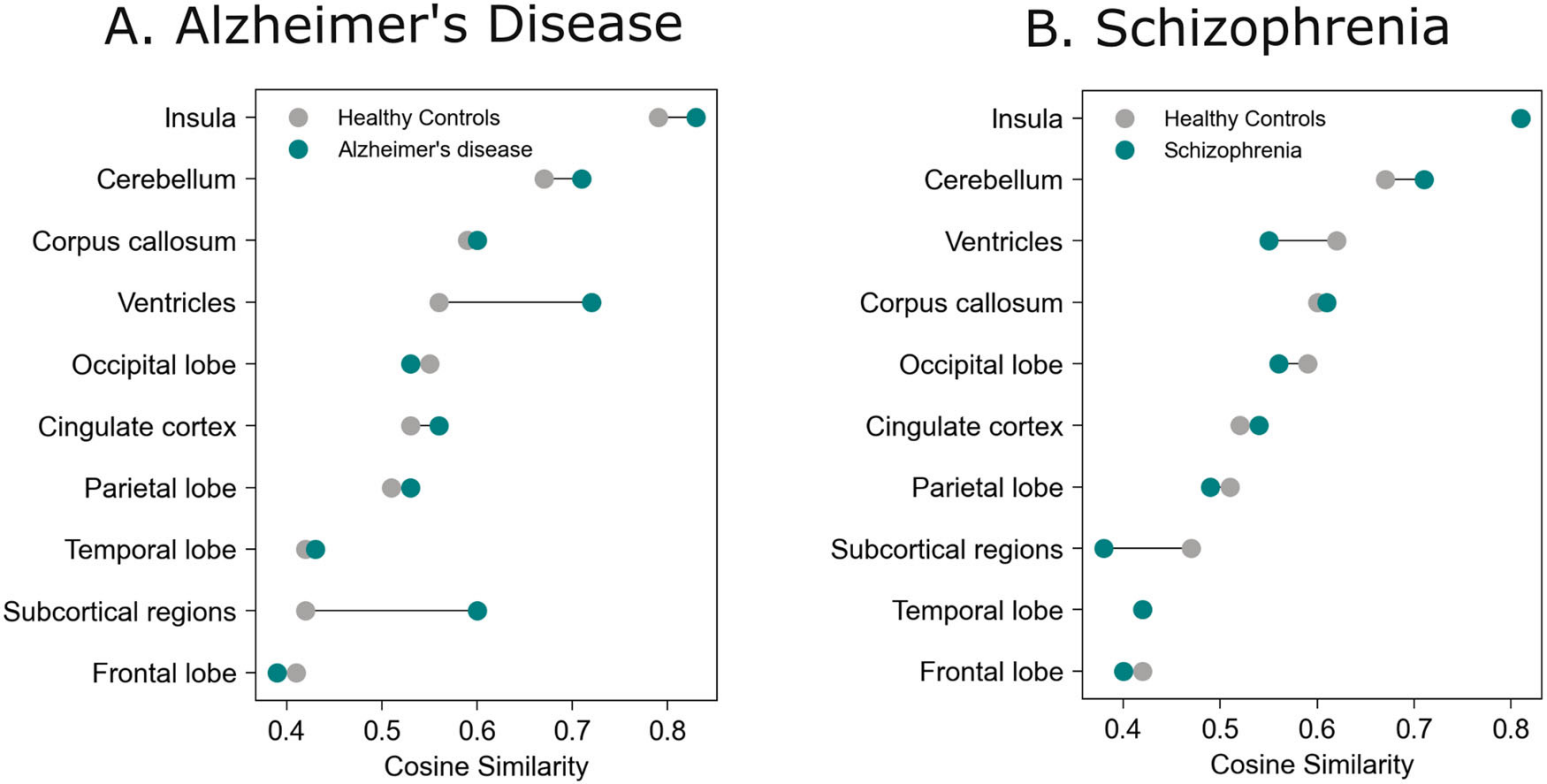
Within-group mean pairwise cosine similarity for high-level brain regions. **A** Alzheimer’s disease patients exhibited greater homogeneity in brain morphology compared to healthy controls, with the highest similarity observed in the ventricles and subcortical regions. **B** In contrast, Schizophrenia patients were slightly more heterogeneous than their respective controls, with the greatest differences in cosine similarity found in the ventricles and subcortical regions.

Across all ROIs, AD patients were more homogeneous (Mean CS = 0.54, SD = 0.22) than their corresponding HC (Mean CS = 0.31, SD = 0.25). Higher homogeneity in patients was found for the ventricles (Mean CS_HC_ = 0.56, SD = 0.24); Mean CS_AD_ = 0.72, SD = 0.20) and subcortical regions (Mean CS_HC_ = 0.42, SD = 0.20; Mean CS_AD_ = 0.60, SD = 0.20). Conversely, SZ patients were slightly more heterogeneous (Mean CS = 0.32, SD = 0.15) than HC (Mean CS = 0.40, SD = 0.17) across all ROIs. This was most visible in the ventricles (CS_HC_ = 0.62, SD = 0.23; CS_SZ_ = 0.55, SD = 0.22) and subcortical regions (CS_HC_ = 0.47, SD = 0.21); CS_SZ_ = 0.38, SD = 0.20) (Fig. 7, see Supplementary Materials for detailed results).

## Discussion

The recent widespread interest in personalised psychiatry and neurology, combined with the increasing availability of large imaging datasets, has propelled a renewed interest in normative modelling [12, 15, 78]. This approach involves first modelling a feature of interest in a reference cohort, usually healthy controls, and then mapping patients against this model. It is then possible to calculate the deviation from the reference cohort, also known as the ‘norm’, for each individual patient. Interesting findings include evidence that a large proportion of psychiatric and neurological patients fall within the normative range for brain morphology [16, 17], and that there is high degree of heterogeneity within a diagnosis [6, 18, 19] and overlap between diagnoses [18]. These results are consistent with the current understanding of mental health disorders as dimensional constructs [79], and challenge the premise of the widely used case-control design.

An important next step is to further explore ‘deviation from the norm’ as a potential biomarker. This might involve, for example, estimating deviation from the norm in several clinical populations, investigating what drives extreme deviations within a diagnosis and how such deviations relate to clinical outcomes or other relevant variables such as genetic risk and cognitive performance. On the one hand, the typical output of a normative model is intuitive, informative and easy to use in further statistical analyses. On the other hand, the development of normative models may not be feasible for most researchers due to lack of technical expertise, adequate datasets or/and computational resources. Neurofind was developed to bridge this gap and make normative modelling accessible to the wider research community. A number of strategies were adopted to maximise the usability and utility of the tool [80]. Firstly, Neurofind was developed as a *user-friendly* web-based tool with minimal technical requirements. The tool only requires a good internet connection, good quality MRI scans in the appropriate format and key information about the images from the user. This makes the tool suitable for non-experts in imaging and/or normative modelling. Secondly, Neurofind requires no prior *processing* of the images from the user. The only preprocessing necessary is to convert the scan into the required format (i.e., NIfTI), which is already standard practice in the field and accessible to most researchers. Thirdly, Neurofind can be applied to *any adult population*. By using whole-brain data across a large age range, Neurofind can be used to investigate brain morphology of psychiatric diagnoses. This differs from existing tools that are targeted at neurological disorders and therefore focus on relevant metrics and/or narrower age range such as gray matter of sub-cortical structures to detect AD or white matter tracts to detect multiple sclerosis. Finally, the tool provides *two complementary normative metrics*. Interest in anomaly detection and brain age is growing rapidly in translational brain sciences. Making these models easily accessible to the wider research community will hopefully accelerate the field of normative modelling in psychiatric and neurological research. Notably, both metrics are transdiagnostic and derived from whole-brain data, allowing Neurofind to be applied to a group of individuals of interest without making any assumptions about their diagnoses. This will hopefully facilitate research on unique and shared brain morphology between diagnoses for example, as encouraged by dimensional approaches to psychopathology such as the Research Domain Criteria [79].

To illustrate the potential of Neurofind to investigate different aspects of any disorder of interest, we provided examples of possible analyses in AD and SZ. As expected, we found that AD patients have larger overall deviations from the normative brain morphology than comparable controls. Deviations were more pronounced in the ventricles and limbic-temporal regions. Alterations in these regions are well documented in literature on AD [81, 82]. In addition, the magnitude of the deviation from the norm for two of these regions - hippocampus and middle temporal gyrus - were negatively associated severity of dementia, such that patients with larger deviations from the norm presented with worse symptoms. We also showed that AD patients tended to be more homogeneous than comparable controls. This is an interesting finding that highlights the well-established morphological changes in AD. Consistent with the results from the group comparisons, homogeneity between patients was most pronounced in the ventricles and subcortical regions, which further emphasises the role of these brain regions in AD. In the analysis of brain age, AD patients had an older-appearing brain than comparable healthy controls consistent with the literature [46]. The results for SZ were not as clear cut. This is not unexpected given that, compared with AD, SZ has less clear morphological markers [83]. This may explain why the overall deviation from the norm was not statistically different from comparable controls, while specific regions namely the hippocampus and putamen were. These findings are also consistent with well documented brain alterations in SZ. Abnormal hippocampal volume in SZ is thought to be implicated in cognitive [84–86] and emotional processing [87, 88] deficits as well as in the dysregulation of the hypothalamic-pituitary-adrenal axis [89]. Similarly, the pallidum also plays a central role in the dopaminergic dysfunction in SZ [90]. Finally, SZ patients tended to be more heterogenous than controls across all regions and most of the high-level regions. Inter-individual variation in SZ is well-known but only recently have there been systematic efforts to quantify it [2, 91–93]. SZ patients also showed signs of accelerated ageing consistent with previous studies [46]. Taken collectively, these results indicate that Neurofind is capable of identifying meaningful deviations from the norm and can be a useful tool to parse heterogeneity in brain-based disorders. The less clear-cut results for schizophrenia are consistent with the literature on normative modelling, showing that only a small portion of patients fall outside the norm and that patients are highly heterogenous in how they deviate from the expected [16, 18, 94]. Our aim with these exemplar analyses was to illustrate how Neurofind can be used to explore psychiatric and neurological disorders. There are many ways in which Neurofind can be explored further. For example, clustering patients based on region-level deviations, identifying differences in clinical presentation between within the norm and extreme patients, stratifying patients in terms of longitudinal outcomes, or investigate the association of brain deviations and behavioural/cognitive/health traits in healthy controls, such as BMI, substance use, cognitive performance. We encourage researchers to always use Neurofind in patients in combination with a group of comparable healthy controls, as shown in our examples, to control for biases specific to their sample. Inferences in patients scores should then be made in relation this comparison group.

The development of a practical tool for the wider research community, such as Neurofind, comes with a number of limitations and assumptions. Neurofind was designed to make normative modelling accessible and therefore it is not customisable. Advanced users may prefer to have more control over certain aspects of the data and/or model training. In addition, Neurofind was developed to provide general purpose and transdiagnostic metrics that can be used across psychiatric and neurological research. It was not developed to identify specific disorders or predict clinical outcomes in a real-world clinical setting. To maintain simplicity and interpretability of the findings, the brain age model in Neurofind did not take its statistical dependency on chronological age into account. Because of this so-called ‘regression to the mean’ or ‘age bias’, the ages of younger subjects tend to be overestimated, while the ages of older subjects tend to be underestimated. We recommend that any further analysis of brain age uses age as a covariate or addresses this issue in another way [95]. Neuroharmony, the method used to minimise the impact of scanner-related bias, relies on a learned association between image quality metrics and needed corrections. For Neuroharmony to work effectively, it is necessary that the quality metrics of the images fall within a certain range; conversely, Neuroharmony may not be able to predict the required adjustments if the quality metrics are unusual (for more information see [56]). Similarly, Neurofind may not be suitable for analysis of subjects that are very different from our training range, i.e., outside of ages 20–80, non-white ethnicities, or MRI scans acquired with fields of strength considerably lower or higher than 1.5 T or 3 T. Finally, it is important to note that Neurofind is a research tool, so results should not be used to make clinical decisions.

In conclusion, Neurofind is a new freely available tool that aims to facilitate research in normative modelling in psychiatry and neurology. It is aimed at the wider community and non-expert researchers who wish to use this approach in their research. It relies on sound methods previously published on data harmonisation, brain age algorithms and deep learning. We have presented exemplar applications showing that Neurofind can produce interpretable and meaningful results in line with the literature on AD and SZ. We have also provided a how-to guide that illustrates the use Neurofind in four simple steps. Neurofind can be accessed via www.neurofind.ai and relevant publications and materials that describe the scanner harmonization, AAE and brain age methods in detail are also available [56, 59, 60].

## Supplementary information

Supplementary materials

## Data Availability

All data used in this study were obtained from pre-existing datasets with varying access procedures. Researchers can refer to the individual dataset links below for further details: AIBL (https://aibl.csiro.au/adni/index.html), COBRE (https://fcon_1000.projects.nitrc.org/indi/retro/cobre.html), Human Connectome Project Aging (https://www.humanconnectome.org/study/hcp-lifespan-aging/data-releases), Human Connectome Project Young Adults (https://www.humanconnectome.org/study/hcp-young-adult/data-releases), IXI (http://brain-development.org/ixi-dataset/), MCIC (www.mrn.org), UK Biobank (https://www.ukbiobank.ac.uk/enable-your-research).

## Code availability

The code for model development is available at https://github.com/MLMH-Lab/neurofind_ai.
