# Supplementary material for: Neurofind: using deep learning to make individualised inferences in brain-based disorders
Source: Transl Psychiatry. 2025 Feb 27;15:69. doi: 10.1038/s41398-025-03290-x (PMC11868583; doi:10.1038/s41398-025-03290-x)
Supplement: Supplementary file 1 — Supplementary materials [file 41398_2025_3290_MOESM1_ESM.docx]

**Neurofind: Using deep learning to make individualised inferences in brain-based disorders**

Supplementary Materials

Table of Contents

[1. Methods 1](#_Toc179965577)

[Table S1. Image acquisition parameters 1](#_Toc179965578)

[Table S2. List of FreeSurfer regions 1](#_Toc179965579)

[Figure S1. Scanner harmonisation. 3](#_Toc179965580)

[1.1. AAE structure and training 3](#_Toc179965581)

[Table S3. Final model configuration. 3](#_Toc179965582)

[Figure S2. Distribution of ROI and whole brain scaled reconstruction errors in the train set. 4](#_Toc179965583)

[Table S4. Desikan-Killiany ROIs grouping into brain regions. 5](#_Toc179965584)

[2. Results 5](#_Toc179965585)

[Table S5. Normality testing and group comparisons for Overall OIS. 5](#_Toc179965586)

[Table S6. Normality testing and group comparisons for Brain Age Gap. 5](#_Toc179965587)

[Table S7. Comparison of ROI-level OIS between in Alzheimer’s disease and healthy controls. 5](#_Toc179965588)

[Table S8. Comparison of ROI-level OIS between in Schizophrenia and healthy controls. 8](#_Toc179965589)

[Table S9. Shapiro’s Wilk test for positive, negative and dementia symptoms. 10](#_Toc179965590)

[Table S10. Correlation between statistically significant ROI between Alzheimer’s disease and healthy controls and MMSE. 10](#_Toc179965591)

[Table S11. Correlation between statistically significant ROI between Schizophrenia and healthy controls and positive/ negative symptoms. 10](#_Toc179965592)

[Table S12. Mean cosine similarity of deviation scores amongst patients and controls. 10](#_Toc179965593)

# 1. Methods

# Table S1. Image acquisition parameters

|  | Final N | Manufacturer | Field Intensity (T) | TE (ms) | TR (ms) | Flip angle (°) | Voxel size (mm) | Matrix dimension |
| --- | --- | --- | --- | --- | --- | --- | --- | --- |
| HCP | 599 | Siemens | 3 | 2.14 | 2400 | 8 | 0.7x0.7x 0.7 | 320x320x256 |
| HCP Aging | 458 | Siemens | 3 | 1.8/ 3.6/ 5.4/ 7.2 | 2500 | 8 | 0.8x0.8x0.8 | 320x300x208 |
| BIOBANK S01 | 1462 | Siemens | 3 | 2.01 | 2000 | 8 | 1x1x1 | 208x256x256 |
| BIOBANK S02 | 349 | Siemens | 3 | 2.01 | 2000 | 8 | 1x1x1 | 208x256x256 |
| IXI S01 | 293 | Philips | 3 | 4.6 | 9.6 | 8 | 1x1x1.2 | 256x256x130 |
| IXI S02 | 165 | Philips | 1.5 | 4.6 | 9.8 | 8 | 1x1x1.2 | 256x256x150 |
| IXI S03 | 36 | GE | 1.5 | 2.4 | 5.8 | 20 | 1x1x1.2 | 256x256x146 |
| AIBL | 122 | Siemens | 1.5 | 2.98 | 2300 | 9 | 1x1x1.2 | 240x256x160 |
| MCIC S01 | 64 | Siemens | 1.5 | 4.76 | 12 | 20 | 0.625x0.625x1.5 | 256x256x128 |
| MCIC S02 | 40 | Siemens | 3 | 3.79 | 2530 | 7 | 0.625x0.625x1.5 | 256x256x128 |
| MCIC S03 | 52 | Siemens | 1.5 | 4.76 | 12 | 20 | 0.625x0.625x1.5 | 256x256x128 |
| COBRE | 86 | Siemens | 3 | 3.5 | 2.53 | 7 | 1x1x1 | 256x256x176 |

# Table S2. List of FreeSurfer regions

| Right amygdala | Right precuneus cortex | Left middle temporal gyrus |
| --- | --- | --- |
| Right banks of the superior temporal sulcus | Right putamen | Left nucleus accumbens |
| Right caudal anterior cingulate cortex | Right rostral anterior cingulate cortex | Left pallidum |
| Right caudal middle frontal gyrus | Right rostral middle frontal gyrus | Left paracentral lobule |
| Right caudate | Right superior frontal gyrus | Left parahippocampal gyrus |
| Right cerebellum cortex | Right superior parietal cortex | Left pars opercularis |
| Right cerebellum white matter | Right superior temporal gyrus | Left pars orbitalis |
| Right cuneus cortex | Right supramarginal gyrus | Left pars triangularis |
| Right entorhinal cortex | Right temporal pole | Left pericalcarine |
| Right frontal pole | Right thalamus proper | Left postcentral gyrus |
| Right fusiform gyrus | Right transverse temporal cortex | Left posterior cingulate cortex |
| Right hippocampus | Right ventral diencephalon | Left precentral gyrus |
| Right inferior lateral ventricle | Left amygdala | Left precuneus cortex |
| Right inferior parietal cortex | Left banks of the superior temporal sulcus | Left putamen |
| Right inferior temporal cortex | Left caudal anterior cingulate cortex | Left rostral anterior cingulate cortex |
| Right insula | Left caudal middle frontal gyrus | Left rostral middle frontal gyrus |
| Right isthmus-cingulate cortex | Left caudate | Left superior frontal gyrus |
| Right lateral occipital cortex | Left cerebellum cortex | Left superior parietal cortex |
| Right lateral orbital frontal cortex | Left cerebellum white matter | Left superior temporal gyrus |
| Right lateral ventricle | Left cuneus cortex | Left supramarginal gyrus |
| Right lingual gyrus gyrus | Left entorhinal cortex | Left temporal pole |
| Right medial orbital frontal cortex | Left frontal pole | Left thalamus proper |
| Right middle temporal gyrus | Left fusiform gyrus | Left transverse temporal cortex |
| Right nucleus accumbens | Left hippocampus | Left ventral diencephalon |
| Right pallidum | Left inferior lateral ventricle | Brain stem |
| Right paracentral lobule | Left inferior parietal cortex | Cerebrospinal fluid |
| Right parahippocampal gyrus | Left inferior temporal gyrus | Corpus callosum anterior |
| Right pars opercularis | Left insula | Corpus callosum central |
| Right pars orbitalis | Left isthmus-cingulate cortex | Corpus callosum mid-anterior |
| Right pars triangularis | Left lateral occipital cortex | Corpus callosum mid-posterior |
| Right pericalcarine cortex | Left lateral orbitofrontal | Corpus callosum posterior |
| Right postcentral gyrus | Left lateral ventricle | Fourth ventricle |
| Right posterior cingulate cortex | Left lingual gyrus | Third ventricle |
| Right precentral gyrus | Left medial orbital frontal cortex |  |


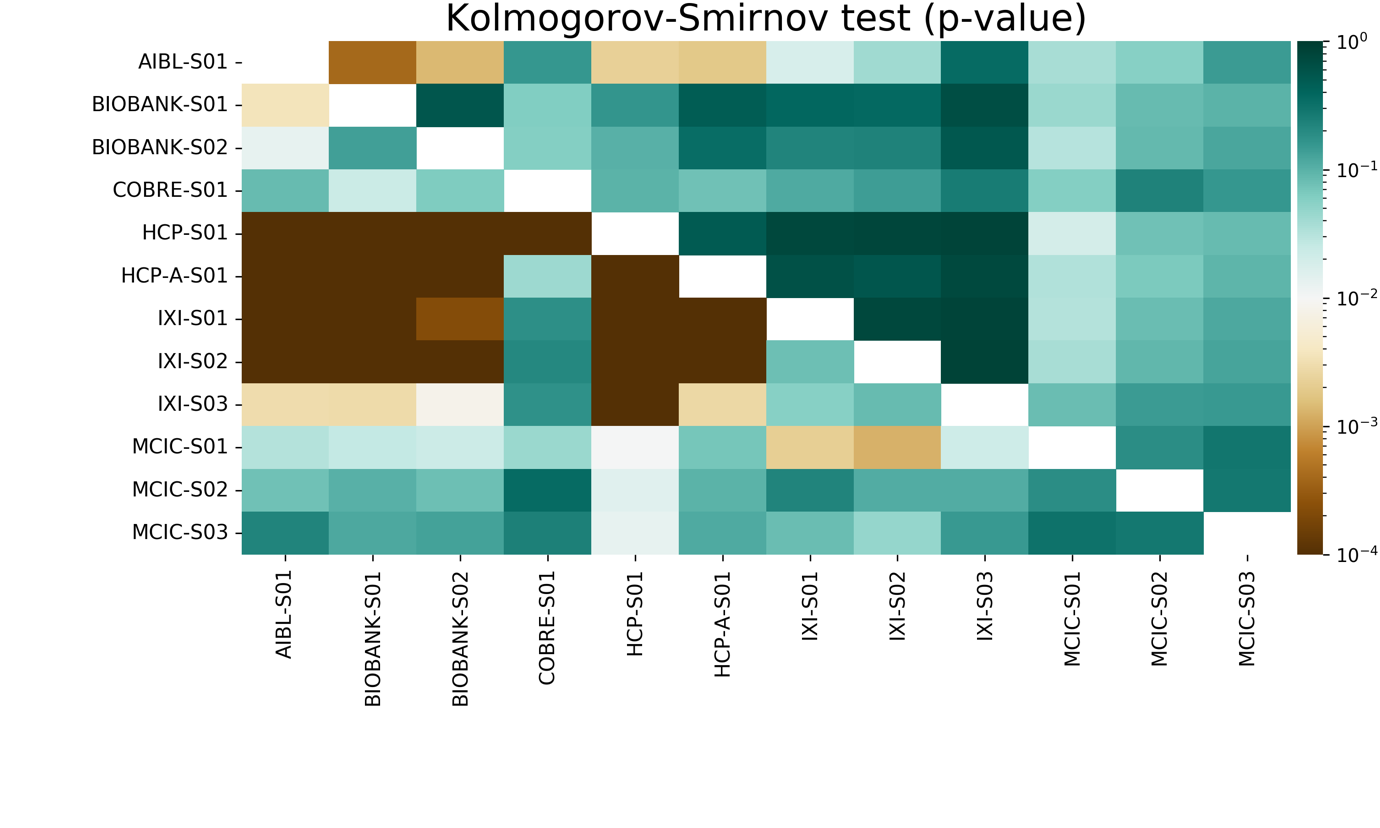


# Figure S1. Scanner harmonisation. To evaluate the efficacy of the harmonization, the nonparametric Kolmogorov-Smirnov two-sample test (K–S test) was applied to the relative volumes of each ROI for each pair of scanners for controls only across all available data (train and clinical samples). The figure shows the median p-values for the K–S test across all ROIs, before and after harmonization with Neuroharmony. The cells under the main diagonal of the matrix represent the K–S test p-values before harmonization, while the values in the top of the main diagonal represent the p-values after the harmonization. Each cell corresponds to a pair of scanners. Cells are coloured as shown on the colour bar. Overall, the magnitude of the p-values increased from pre- to post-harmonisation indicating that the distributions of controls are less distinguishable after harmonisation.

# 1.1. AAE structure and training

The model structure and training follow the same principals as our previous work; for a detailed description please see ^1,2^. The final architecture of the deep AAE comprised of an input layer with 101 neurons, an encoder with one hidden with 25 neurons and a latent code with 5 neurons. The decoder and the discriminator had a similar structure (one hidden layer with 25 neurons). The output layer was the same structure as the input layer (101 neurons). All hidden layers had a leaky ReLU non-linearity. The latent code and the decoder’s output layer had a linear activation function. Before training, the relative brain region volumes (ROI_rel_) were normalised across all the participants in the training set. The normalisation was robust to outliers by subtracting the median value of the relative brain region volume and then scaling the data according to its inter-quartile range. Normalisation was done independently for each brain region. The same metrics (median and interquartile range) from the train set were used to normalise the clinical datasets. To further account for the effect of demographics variables, age and sex were added to the model such that the AAE was conditioned to learn a latent representation whilst taking into account this information. The weights of the network were initialised with standard Glorot initialization (Glorot & Bengio, 2010). AAE weights were then optimised by minimising the mean squared value of its reconstruction error using the gradient descent-based optimizer Adam with a batch size of 64. The learning rate was adjusted using cyclical learning with a base learning rate of 0.00002, a maximum learning rate of 0.005, and a basic triangular shape with an amplitude decaying (gamma = 0.038). The number of hidden layers, the number of neurons in each hidden layer, the number of neurons the latent code, batch size and gamma were optimised using grid search in the train set with 75% of the data for training and the remaining 25% for validation of the hyperparameters. The best configuration was used to train the entire training set.

# Table S3. Final model configuration.

| Main autoencoder layers and neurons | [101, 25, 5, 25 101] |
| --- | --- |
| Activation function | Leaky ReLU / Linear |
| Weights initialization | Glorot |
| Optimiser | Adam |
| Batch size | 64 |
| Learning rate | 2e−5 |

**
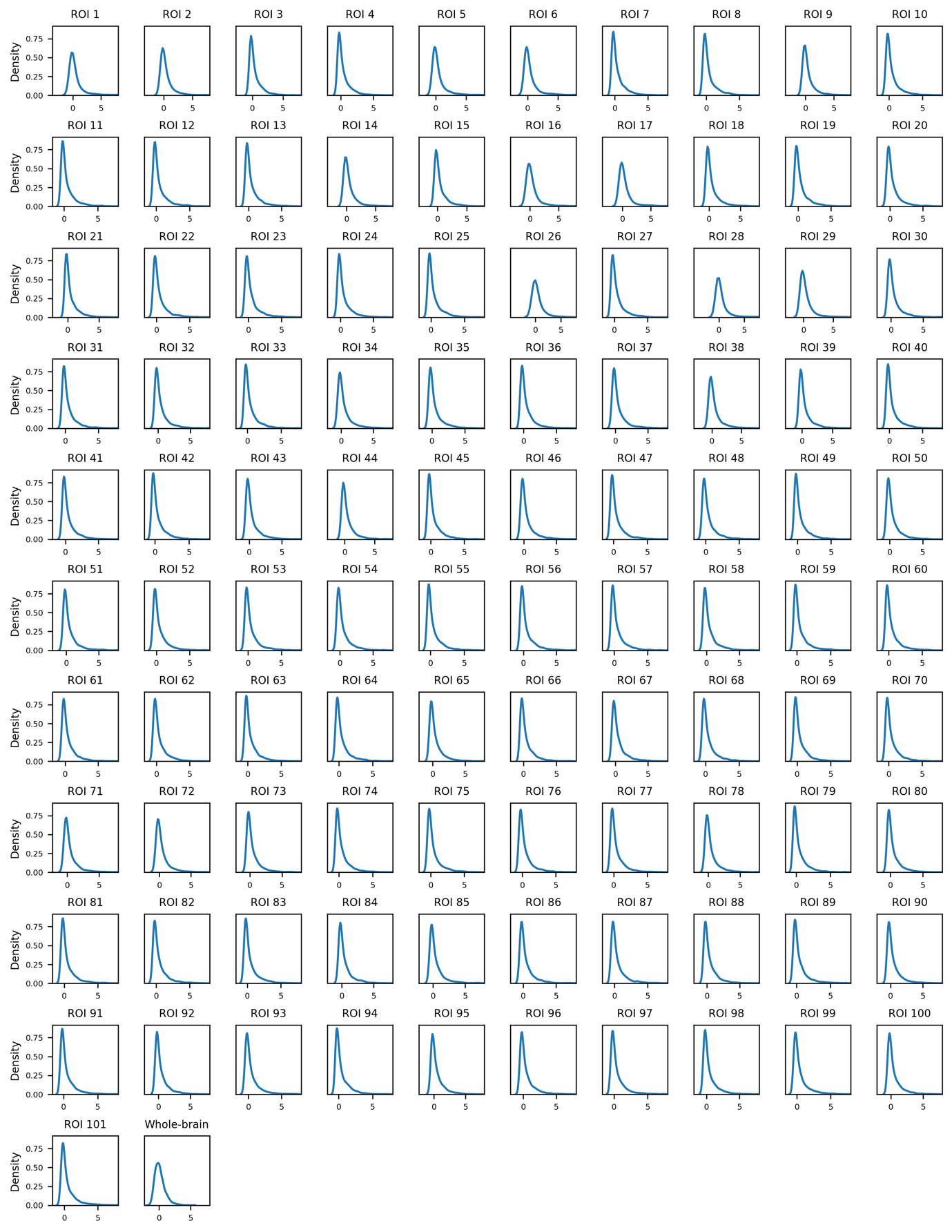
**

Figure S2. Distribution of ROI and whole brain scaled reconstruction errors in the train set. ROI 1: 3rd-Ventricle; ROI 2: 4th-Ventricle; ROI 3: Brain-Stem; ROI 4: CC_Anterior; ROI 5: CC_Central; ROI 6: CC_Mid_Anterior; ROI 7: CC_Mid_Posterior; ROI 8: CC_Posterior; ROI 9: CSF; ROI 10: Left-Accumbens-area; ROI 11: Left-Amygdala; ROI 12: Left-Caudate; ROI 13: Left-Cerebellum-Cortex; ROI 14: Left-Cerebellum-White-Matter; ROI 15: Left-Hippocampus; ROI 16: Left-Inf-Lat-Vent; ROI 17: Left-Lateral-Ventricle; ROI 18: Left-Pallidum; ROI 19: Left-Putamen; ROI 20: Left-Thalamus-Proper; ROI 21: Left-VentralDC; ROI 22: Right-Accumbens-area;

ROI 23: Right-Amygdala; ROI 24: Right-Caudate; ROI 25: Right-Cerebellum-Cortex; ROI 26: Right-Cerebellum-White-Matter; ROI 27: Right-Hippocampus; ROI 28: Right-Inf-Lat-Vent; ROI 29: Right-Lateral-Ventricle; ROI 30: Right-Pallidum; ROI 31: Right-Putamen; ROI 32: Right-Thalamus-Proper; ROI 33: Right-VentralDC; ROI 34: lh_bankssts_volume; ROI 35: lh_caudalanteriorcingulate_volume; ROI 36: lh_caudalmiddlefrontal_volume; ROI 37: lh_cuneus_volume; ROI 38: lh_entorhinal_volume; ROI 39: lh_frontalpole_volume; ROI 40: lh_fusiform_volume; ROI 41: lh_inferiorparietal_volume; ROI 42: lh_inferiortemporal_volume; ROI 43: lh_insula_volume; ROI 44: lh_isthmuscingulate_volume; ROI 45: lh_lateraloccipital_volume; ROI 46: lh_lateralorbitofrontal_volume; ROI 47: lh_lingual_volume; ROI 48: lh_medialorbitofrontal_volume; ROI 49: lh_middletemporal_volume; ROI 50: lh_paracentral_volume; ROI 51: lh_parahippocampal_volume; ROI 52: lh_parsopercularis_volume; ROI 53: lh_parsorbitalis_volume; ROI 54: lh_parstriangularis_volume; ROI 55: lh_pericalcarine_volume; ROI 56: lh_postcentral_volume; ROI 57: lh_posteriorcingulate_volume; ROI 58: lh_precentral_volume; ROI 59: lh_precuneus_volume; ROI 60: lh_rostralanteriorcingulate_volume; ROI 61: lh_rostralmiddlefrontal_volume; ROI 62: lh_superiorfrontal_volume; ROI 63: lh_superiorparietal_volume; ROI 64: lh_superiortemporal_volume; ROI 65: lh_supramarginal_volume; ROI 66: lh_temporalpole_volume; ROI 67: lh_transversetemporal_volume; ROI 68: rh_bankssts_volume; ROI 69: rh_caudalanteriorcingulate_volume; ROI 70: rh_caudalmiddlefrontal_volume; ROI 71: rh_cuneus_volume; ROI 72: rh_entorhinal_volume; ROI 73: rh_frontalpole_volume; ROI 74: rh_fusiform_volume; ROI 75: rh_inferiorparietal_volume; ROI 76: rh_inferiortemporal_volume; ROI 77: rh_insula_volume; ROI 78: rh_isthmuscingulate_volume; ROI 79: rh_lateraloccipital_volume; ROI 80: rh_lateralorbitofrontal_volume; ROI 81: rh_lingual_volume; ROI 82: rh_medialorbitofrontal_volume; ROI 83: rh_middletemporal_volume; ROI 84: rh_paracentral_volume; ROI 85: rh_parahippocampal_volume; ROI 86: rh_parsopercularis_volume; ROI 87: rh_parsorbitalis_volume; ROI 88: rh_parstriangularis_volume; ROI 89: rh_pericalcarine_volume; ROI 90: rh_postcentral_volume; ROI 91: rh_posteriorcingulate_volume; ROI 92: rh_precentral_volume; ROI 93: rh_precuneus_volume; ROI 94: rh_rostralanteriorcingulate_volume; ROI 95: rh_rostralmiddlefrontal_volume; ROI 96: rh_superiorfrontal_volume; ROI 97: rh_superiorparietal_volume; ROI 98: rh_superiortemporal_volume; ROI 99: rh_supramarginal_volume; ROI 100: rh_temporalpole_volume; ROI 101: rh_transversetemporal_volume.

# Table S4. Desikan-Killiany ROIs grouping into brain regions.

| Brain Region | ROIs |
| --- | --- |
| Cerebellum | Cerebellum cortex |
| Cingulate Cortex | Rostral anterior cingulate, caudal anterior cingulate, posterior cingulate, isthmus cingulate |
| Corpus Callosum | Corpus callosum |
| Frontal Lobe | Superior frontal, caudal middle frontal, rostral middle frontal, parsopercularis, parsorbitalis, parstriangularis, lateral orbitofrontal, medial orbitofrontal, precentral, frontal pole |
| Insula | Insula |
| Occipital Lobe | Lateral occipital, cuneus, lingual, pericalcarine |
| Parietal Lobe | Superior parietal, inferior parietal, supramarginal, postcentral, precuneus |
| Subcortical Structures | Hippocampus, amygdala, caudate, putamen, pallidum, accumbens area, thalamus |
| Temporal Lobe | Superior temporal, middle temporal, inferior temporal, fusiform, transverse temporal, entorhinal, parahippocampal, temporal pole, bankssts |
| Ventricles | Lateral ventricle, inferior lateral ventricle, third ventricle, fourth ventricle |

# 2. Results

# Table S5. Normality testing and group comparisons for Overall OIS.

| Group | SW Patients | SW Patients p | SW HC | SW HC p | Patients Med (IQR) | Controls Med (IQR) | Mann-U | p |
| --- | --- | --- | --- | --- | --- | --- | --- | --- |
| AD | 0.92 | 0.034 | 0.92 | 0.001 | 2.46 (2.50) | 0.91 (1.36) | 1361.0 | 0.000 |
| SZ | 0.84 | 0.000 | 0.97 | 0.047 | 1.38 (1.83) | 1.10 (1.76) | 7657.0 | 0.091 |

SW: Shapiro Wilk’s test; HC: healthy controls; Med: median; interquartile range; Mann-U; Mann-Whitney U test.

# Table S6. Normality testing and group comparisons for Brain Age Gap.

| Group | SW Patients | SW Patients *p* | SW HC | SW HC *p* | Patients M (SD) | Controls M (SD) | Ind. T-test | *p* |
| --- | --- | --- | --- | --- | --- | --- | --- | --- |
| AD | 1.0 | 0.997 | 0.98 | 0.598 | -0.12 ± 0.43 | -0.61 ± 0.38 | 5.45 | 0.000 |
| SZ | 1.0 | 0.653 | 0.98 | 0.250 | 0.77 ± 0.50 | 0.57 ± 0.44 | 3.22 | 0.001 |

SW: Shapiro Wilk’s test; HC: healthy controls; M: mean; SD: standard deviation.

# Table S7. Comparison of ROI-level OIS between in Alzheimer’s disease and healthy controls.

| ROI | SW Patients | SW Patients *p* | SW HC | SW HC *p* | Patients Med (IQR) | HC Med (IQR) | Mann-U | Adj *p* |
| --- | --- | --- | --- | --- | --- | --- | --- | --- |
| 3rd-Ventricle | 0.89 | 0.007 | 0.52 | 0.000 | 3.57 (5.62) | 0.92 (4.29) | 1041 | 0.373 |
| 4th-Ventricle | 0.77 | 0.000 | 0.55 | 0.000 | -0.10 (0.82) | 0.06 (1.14) | 756 | 0.771 |
| Brain-Stem | 0.67 | 0.000 | 0.80 | 0.000 | 0.44 (1.74) | 0.24 (1.23) | 909 | 0.850 |
| CC_Anterior | 0.66 | 0.000 | 0.72 | 0.000 | -0.12 (1.17) | 0.08 (0.94) | 842 | 0.998 |
| CC_Central | 0.69 | 0.000 | 0.58 | 0.000 | 0.05 (1.45) | 0.04 (0.83) | 894 | 0.913 |
| CC_Mid_Anterior | 0.71 | 0.000 | 0.43 | 0.000 | 0.41 (0.94) | 0.11 (1.08) | 1005 | 0.558 |
| CC_Mid_Posterior | 0.80 | 0.000 | 0.88 | 0.000 | 0.62 (2.11) | 0.51 (1.88) | 888 | 0.915 |
| CC_Posterior | 0.68 | 0.000 | 0.69 | 0.000 | 0.21 (1.22) | -0.13 (0.85) | 1042 | 0.373 |
| CSF | 0.62 | 0.000 | 0.35 | 0.000 | 1.20 (2.19) | 0.16 (1.15) | 1074 | 0.291 |
| Left-Accumbens-area | 0.76 | 0.000 | 0.79 | 0.000 | 0.59 (2.79) | 0.05 (1.17) | 1048 | 0.373 |
| Left-Amygdala | 0.88 | 0.003 | 0.63 | 0.000 | 2.77 (3.47) | 0.17 (2.12) | 1196 | **0.022** |
| Left-Caudate | 0.77 | 0.000 | 0.31 | 0.000 | 0.05 (1.14) | 0.25 (1.36) | 776 | 0.861 |
| Left-Cerebellum-Cortex | 0.55 | 0.000 | 0.70 | 0.000 | -0.10 (0.82) | 0.21 (1.93) | 654 | 0.408 |
| Left-Cerebellum-White-Matter | 0.77 | 0.000 | 0.80 | 0.000 | 0.04 (1.19) | 0.11 (1.77) | 875 | 0.915 |
| Left-Hippocampus | 0.94 | 0.075 | 0.80 | 0.000 | 3.89 (4.03) | 0.21 (1.40) | 1476 | **0.000** |
| Left-Inf-Lat-Vent | 0.94 | 0.107 | 0.73 | 0.000 | 13.14 (13.26) | 0.93 (2.52) | 1519 | **0.000** |
| Left-Lateral-Ventricle | 0.73 | 0.000 | 0.61 | 0.000 | 2.82 (6.55) | 0.25 (2.19) | 1160 | 0.063 |
| Left-Pallidum | 0.78 | 0.000 | 0.68 | 0.000 | 0.50 (1.92) | -0.11 (1.41) | 1095 | 0.202 |
| Left-Putamen | 0.79 | 0.000 | 0.74 | 0.000 | 0.34 (2.21) | 0.08 (1.20) | 985 | 0.647 |
| Left-Thalamus-Proper | 0.82 | 0.000 | 0.71 | 0.000 | 0.92 (1.88) | 0.47 (1.71) | 879 | 0.915 |
| Left-VentralDC | 0.72 | 0.000 | 0.76 | 0.000 | 0.19 (1.71) | 0.11 (1.09) | 866 | 0.934 |
| Right-Accumbens-area | 0.92 | 0.039 | 0.59 | 0.000 | 0.93 (2.23) | 0.13 (1.60) | 1154 | 0.063 |
| Right-Amygdala | 0.90 | 0.010 | 0.62 | 0.000 | 2.60 (6.05) | 0.15 (1.65) | 1246 | **0.000** |
| Right-Caudate | 0.74 | 0.000 | 0.27 | 0.000 | -0.01 (1.13) | 0.21 (1.48) | 742 | 0.758 |
| Right-Cerebellum-Cortex | 0.69 | 0.000 | 0.82 | 0.000 | 0.17 (1.36) | 0.36 (1.97) | 714 | 0.711 |
| Right-Cerebellum-White-Matter | 0.77 | 0.000 | 0.67 | 0.000 | 0.06 (1.26) | 0.18 (1.62) | 850 | 0.983 |
| Right-Hippocampus | 0.94 | 0.130 | 0.79 | 0.000 | 5.18 (4.53) | 0.16 (1.63) | 1425 | **0.000** |
| Right-Inf-Lat-Vent | 0.89 | 0.005 | 0.60 | 0.000 | 4.73 (14.11) | 0.37 (1.73) | 1382 | **0.000** |
| Right-Lateral-Ventricle | 0.73 | 0.000 | 0.55 | 0.000 | 2.59 (5.07) | 0.08 (1.29) | 1118 | 0.131 |
| Right-Pallidum | 0.76 | 0.000 | 0.68 | 0.000 | 0.46 (1.90) | -0.11 (0.88) | 1056 | 0.369 |
| Right-Putamen | 0.80 | 0.000 | 0.58 | 0.000 | 0.57 (1.90) | 0.17 (1.19) | 1031 | 0.404 |
| Right-Thalamus-Proper | 0.69 | 0.000 | 0.49 | 0.000 | 0.38 (1.79) | 0.21 (1.49) | 970 | 0.711 |
| Right-VentralDC | 0.86 | 0.001 | 0.79 | 0.000 | 0.96 (3.01) | 0.26 (1.56) | 946 | 0.758 |
| lh_bankssts_volume | 0.85 | 0.001 | 0.77 | 0.000 | 1.22 (2.62) | 0.21 (0.90) | 1153 | 0.063 |
| lh_caudalanteriorcingulate_volume | 0.80 | 0.000 | 0.68 | 0.000 | -0.14 (1.10) | 0.09 (1.38) | 646 | 0.381 |
| lh_caudalmiddlefrontal_volume | 0.75 | 0.000 | 0.40 | 0.000 | -0.10 (0.73) | -0.14 (0.67) | 936 | 0.767 |
| lh_cuneus_volume | 0.59 | 0.000 | 0.56 | 0.000 | -0.04 (1.39) | 0.02 (1.23) | 906 | 0.861 |
| lh_entorhinal_volume | 0.84 | 0.000 | 0.77 | 0.000 | 0.45 (2.08) | 0.12 (1.76) | 1012 | 0.515 |
| lh_frontalpole_volume | 0.35 | 0.000 | 0.51 | 0.000 | 0.18 (0.92) | 0.23 (1.14) | 848 | 0.992 |
| lh_fusiform_volume | 0.77 | 0.000 | 0.74 | 0.000 | 0.38 (1.63) | 0.05 (0.91) | 997 | 0.591 |
| lh_inferiorparietal_volume | 0.72 | 0.000 | 0.79 | 0.000 | 1.48 (3.05) | 0.04 (1.29) | 1185 | **0.040** |
| lh_inferiortemporal_volume | 0.80 | 0.000 | 0.64 | 0.000 | 1.24 (3.26) | -0.07 (0.79) | 1245 | **0.000** |
| lh_insula_volume | 0.78 | 0.000 | 0.63 | 0.000 | 0.55 (2.09) | -0.00 (1.29) | 961 | 0.712 |
| lh_isthmuscingulate_volume | 0.77 | 0.000 | 0.76 | 0.000 | -0.02 (1.62) | 0.32 (1.15) | 764 | 0.813 |
| lh_lateraloccipital_volume | 0.84 | 0.000 | 0.59 | 0.000 | 0.90 (2.78) | 0.27 (1.40) | 1036 | 0.381 |
| lh_lateralorbitofrontal_volume | 0.73 | 0.000 | 0.77 | 0.000 | -0.15 (1.33) | 0.22 (1.12) | 792 | 0.915 |
| lh_lingual_volume | 0.81 | 0.000 | 0.78 | 0.000 | 0.28 (1.41) | 0.21 (1.62) | 842 | 0.998 |
| lh_medialorbitofrontal_volume | 0.90 | 0.011 | 0.68 | 0.000 | 0.24 (0.60) | -0.07 (1.13) | 965 | 0.711 |
| lh_middletemporal_volume | 0.69 | 0.000 | 0.63 | 0.000 | 0.79 (2.32) | 0.02 (0.89) | 1097 | 0.202 |
| lh_paracentral_volume | 0.64 | 0.000 | 0.60 | 0.000 | 0.38 (1.11) | 0.00 (1.35) | 916 | 0.824 |
| lh_parahippocampal_volume | 0.82 | 0.000 | 0.61 | 0.000 | 0.25 (1.82) | -0.17 (0.72) | 1055 | 0.370 |
| lh_parsopercularis_volume | 0.69 | 0.000 | 0.75 | 0.000 | 0.48 (0.81) | -0.09 (1.02) | 1066 | 0.322 |
| lh_parsorbitalis_volume | 0.66 | 0.000 | 0.73 | 0.000 | 0.05 (1.03) | 0.17 (1.75) | 882 | 0.915 |
| lh_parstriangularis_volume | 0.41 | 0.000 | 0.57 | 0.000 | -0.05 (0.65) | 0.04 (1.05) | 719 | 0.711 |
| lh_pericalcarine_volume | 0.78 | 0.000 | 0.57 | 0.000 | 0.62 (1.18) | 0.27 (2.54) | 885 | 0.915 |
| lh_postcentral_volume | 0.71 | 0.000 | 0.61 | 0.000 | -0.03 (0.75) | 0.31 (1.86) | 720 | 0.711 |
| lh_posteriorcingulate_volume | 0.76 | 0.000 | 0.54 | 0.000 | 0.15 (1.07) | -0.17 (0.50) | 1057 | 0.369 |
| lh_precentral_volume | 0.84 | 0.000 | 0.84 | 0.000 | -0.14 (0.74) | 0.29 (1.35) | 723 | 0.723 |
| lh_precuneus_volume | 0.83 | 0.000 | 0.66 | 0.000 | 0.73 (1.97) | 0.30 (1.23) | 1035 | 0.381 |
| lh_rostralanteriorcingulate_volume | 0.55 | 0.000 | 0.68 | 0.000 | 0.08 (0.69) | -0.14 (0.83) | 830 | 0.973 |
| lh_rostralmiddlefrontal_volume | 0.69 | 0.000 | 0.53 | 0.000 | -0.05 (1.10) | 0.07 (0.92) | 811 | 0.915 |
| lh_superiorfrontal_volume | 0.67 | 0.000 | 0.71 | 0.000 | -0.19 (0.61) | -0.21 (0.82) | 882 | 0.915 |
| lh_superiorparietal_volume | 0.59 | 0.000 | 0.72 | 0.000 | 0.39 (1.10) | 0.11 (1.43) | 933 | 0.767 |
| lh_superiortemporal_volume | 0.70 | 0.000 | 0.69 | 0.000 | 0.18 (1.34) | 0.05 (1.18) | 968 | 0.711 |
| lh_supramarginal_volume | 0.57 | 0.000 | 0.73 | 0.000 | 0.41 (1.93) | -0.21 (0.68) | 1152 | 0.063 |
| lh_temporalpole_volume | 0.85 | 0.001 | 0.71 | 0.000 | 0.08 (0.90) | -0.05 (0.79) | 943 | 0.758 |
| lh_transversetemporal_volume | 0.69 | 0.000 | 0.66 | 0.000 | 0.08 (0.75) | 0.11 (1.48) | 763 | 0.810 |
| rh_bankssts_volume | 0.79 | 0.000 | 0.79 | 0.000 | 0.24 (1.68) | 0.06 (1.38) | 971 | 0.711 |
| rh_caudalanteriorcingulate_volume | 0.78 | 0.000 | 0.73 | 0.000 | 0.08 (0.98) | -0.16 (0.99) | 962 | 0.711 |
| rh_caudalmiddlefrontal_volume | 0.66 | 0.000 | 0.81 | 0.000 | 0.24 (1.41) | 0.05 (1.13) | 940 | 0.758 |
| rh_cuneus_volume | 0.55 | 0.000 | 0.66 | 0.000 | 0.04 (1.61) | 0.05 (1.76) | 823 | 0.965 |
| rh_entorhinal_volume | 0.33 | 0.000 | 0.77 | 0.000 | -0.19 (0.70) | 0.13 (2.27) | 440 | 0.780 |
| rh_frontalpole_volume | 0.32 | 0.000 | 0.42 | 0.000 | -0.01 (0.74) | -0.03 (1.05) | 820 | 0.953 |
| rh_fusiform_volume | 0.70 | 0.000 | 0.56 | 0.000 | 0.28 (1.34) | 0.12 (1.06) | 944 | 0.758 |
| rh_inferiorparietal_volume | 0.80 | 0.000 | 0.77 | 0.000 | 0.37 (3.60) | -0.03 (0.85) | 1154 | 0.063 |
| rh_inferiortemporal_volume | 0.90 | 0.012 | 0.84 | 0.000 | 1.39 (2.98) | 0.02 (1.00) | 1343 | **0.000** |
| rh_insula_volume | 0.86 | 0.001 | 0.86 | 0.000 | 0.31 (1.73) | 0.44 (1.34) | 880 | 0.915 |
| rh_isthmuscingulate_volume | 0.86 | 0.001 | 0.73 | 0.000 | 0.58 (1.55) | 0.40 (1.57) | 924 | 0.771 |
| rh_lateraloccipital_volume | 0.88 | 0.003 | 0.74 | 0.000 | 0.76 (2.57) | 0.04 (1.33) | 1044 | 0.373 |
| rh_lateralorbitofrontal_volume | 0.85 | 0.001 | 0.78 | 0.000 | 0.73 (1.28) | -0.02 (1.29) | 899 | 0.892 |
| rh_lingual_volume | 0.83 | 0.000 | 0.45 | 0.000 | 0.17 (1.73) | 0.10 (1.17) | 873 | 0.915 |
| rh_medialorbitofrontal_volume | 0.83 | 0.000 | 0.71 | 0.000 | 0.32 (1.27) | 0.03 (0.94) | 964 | 0.711 |
| rh_middletemporal_volume | 0.78 | 0.000 | 0.62 | 0.000 | 1.17 (1.79) | -0.08 (0.96) | 1150 | 0.063 |
| rh_paracentral_volume | 0.80 | 0.000 | 0.66 | 0.000 | -0.12 (1.19) | -0.12 (1.12) | 817 | 0.934 |
| rh_parahippocampal_volume | 0.72 | 0.000 | 0.54 | 0.000 | -0.11 (0.68) | 0.00 (1.05) | 796 | 0.915 |
| rh_parsopercularis_volume | 0.49 | 0.000 | 0.65 | 0.000 | -0.20 (0.39) | -0.03 (0.97) | 697 | 0.647 |
| rh_parsorbitalis_volume | 0.73 | 0.000 | 0.71 | 0.000 | -0.06 (1.01) | -0.04 (1.30) | 878 | 0.915 |
| rh_parstriangularis_volume | 0.78 | 0.000 | 0.69 | 0.000 | 0.01 (0.88) | 0.09 (1.08) | 737 | 0.758 |
| rh_pericalcarine_volume | 0.71 | 0.000 | 0.66 | 0.000 | 0.36 (1.51) | 0.27 (1.83) | 931 | 0.771 |
| rh_postcentral_volume | 0.74 | 0.000 | 0.59 | 0.000 | 0.15 (0.83) | -0.04 (1.33) | 846 | 0.998 |
| rh_posteriorcingulate_volume | 0.61 | 0.000 | 0.75 | 0.000 | 0.07 (1.10) | 0.07 (0.87) | 865 | 0.934 |
| rh_precentral_volume | 0.77 | 0.000 | 0.74 | 0.000 | 0.15 (1.56) | -0.00 (1.04) | 925 | 0.771 |
| rh_precuneus_volume | 0.59 | 0.000 | 0.71 | 0.000 | 0.21 (1.66) | 0.12 (1.38) | 826 | 0.973 |
| rh_rostralanteriorcingulate_volume | 0.77 | 0.000 | 0.66 | 0.000 | -0.11 (0.81) | -0.18 (0.71) | 928 | 0.771 |
| rh_rostralmiddlefrontal_volume | 0.71 | 0.000 | 0.78 | 0.000 | 0.03 (0.82) | -0.08 (0.98) | 880 | 0.915 |
| rh_superiorfrontal_volume | 0.70 | 0.000 | 0.75 | 0.000 | -0.03 (0.75) | -0.05 (0.94) | 962 | 0.711 |
| rh_superiorparietal_volume | 0.62 | 0.000 | 0.78 | 0.000 | 0.20 (1.07) | 0.18 (1.15) | 879 | 0.443 |
| rh_superiortemporal_volume | 0.86 | 0.001 | 0.79 | 0.000 | 0.51 (1.74) | 0.19 (1.23) | 926 | 0.443 |
| rh_supramarginal_volume | 0.78 | 0.000 | 0.79 | 0.000 | 0.13 (1.77) | 0.01 (0.81) | 1014 | 0.429 |
| rh_temporalpole_volume | 0.65 | 0.000 | 0.77 | 0.000 | -0.08 (0.62) | -0.02 (1.09) | 777 | 0.832 |
| rh_transversetemporal_volume | 0.64 | 0.000 | 0.66 | 0.000 | 0.06 (0.81) | -0.07 (1.28) | 872 | 0.841 |

SW: Shapiro Wilk’s test; HC: healthy controls; Med: median; interquartile range; Mann-U; Mann-Whitney U test; Adj: adjusted.

# Table S8. Comparison of ROI-level OIS between in Schizophrenia and healthy controls.

| ROI | SW Patients | SW Patients  *p* | SW HC | SW HC  *p* | Patients Med (IQR) | HC  Med (IQR) | Mann-U | Adj *p* |
| --- | --- | --- | --- | --- | --- | --- | --- | --- |
| 3rd-Ventricle | 0.38 | 0.000 | 0.93 | 0.000 | 0.73 (1.59) | 0.97 (1.88) | 5572 | 0.511 |
| 4th-Ventricle | 0.29 | 0.000 | 0.70 | 0.000 | 0.01 (0.95) | 0.24 (1.07) | 5924 | 0.762 |
| CC_Anterior | 0.75 | 0.000 | 0.75 | 0.000 | 0.04 (0.97) | 0.07 (1.16) | 6014 | 0.847 |
| CC_Central | 0.79 | 0.000 | 0.82 | 0.000 | 0.68 (2.36) | 0.80 (2.66) | 5760 | 0.700 |
| CC_Mid_Anterior | 0.78 | 0.000 | 0.77 | 0.000 | 0.48 (2.11) | 0.78 (2.85) | 5901 | 0.750 |
| CC_Mid_Posterior | 0.64 | 0.000 | 0.73 | 0.000 | -0.01 (1.23) | 0.09 (1.21) | 5908 | 0.750 |
| CC_Posterior | 0.79 | 0.000 | 0.73 | 0.000 | 0.28 (1.53) | 0.03 (1.14) | 7015 | 0.571 |
| Left-Accumbens-area | 0.74 | 0.000 | 0.68 | 0.000 | 0.39 (2.26) | 0.34 (1.75) | 6575 | 0.889 |
| Left-Amygdala | 0.63 | 0.000 | 0.78 | 0.000 | -0.06 (1.08) | -0.05 (1.07) | 6365 | 0.970 |
| Left-Caudate | 0.73 | 0.000 | 0.67 | 0.000 | 0.39 (1.94) | 0.06 (0.87) | 7623 | 0.086 |
| Left-Cerebellum-Cortex | 0.69 | 0.000 | 0.64 | 0.000 | 0.03 (0.93) | -0.03 (0.83) | 6637 | 0.834 |
| Left-Hippocampus | 0.66 | 0.000 | 0.70 | 0.000 | 0.35 (1.71) | -0.08 (0.76) | 8077 | **0.038** |
| Left-Inf-Lat-Vent | 0.49 | 0.000 | 0.72 | 0.000 | 0.04 (1.48) | 0.20 (0.94) | 6134 | 0.916 |
| Left-Lateral-Ventricle | 0.16 | 0.000 | 0.91 | 0.000 | 0.35 (1.52) | 0.35 (1.38) | 5886 | 0.745 |
| Left-Pallidum | 0.76 | 0.000 | 0.70 | 0.000 | 0.44 (1.99) | 0.08 (1.22) | 7147 | 0.401 |
| Left-Putamen | 0.65 | 0.000 | 0.72 | 0.000 | 0.28 (1.54) | -0.02 (0.86) | 7846 | **0.043** |
| Left-Thalamus-Proper | 0.70 | 0.000 | 0.81 | 0.000 | 0.91 (3.37) | 1.50 (3.69) | 5744 | 0.700 |
| Left-VentralDC | 0.71 | 0.000 | 0.69 | 0.000 | 0.10 (1.42) | 0.00 (1.02) | 7034 | 0.548 |
| Right-Accumbens-area | 0.59 | 0.000 | 0.76 | 0.000 | 0.39 (2.01) | 0.02 (1.50) | 7330 | 0.303 |
| Right-Amygdala | 0.65 | 0.000 | 0.55 | 0.000 | -0.01 (1.21) | 0.05 (0.84) | 6176 | 0.916 |
| Right-Caudate | 0.68 | 0.000 | 0.78 | 0.000 | 0.25 (1.46) | 0.09 (1.17) | 6791 | 0.742 |
| Right-Cerebellum-Cortex | 0.63 | 0.000 | 0.81 | 0.000 | 0.25 (1.56) | 0.23 (1.47) | 6763 | 0.742 |
| Right-Hippocampus | 0.57 | 0.000 | 0.65 | 0.000 | 0.30 (1.66) | -0.12 (0.77) | 7381 | **0.045** |
| Right-Inf-Lat-Vent | 0.51 | 0.000 | 0.54 | 0.000 | 0.01 (1.39) | 0.25 (1.36) | 5819 | 0.733 |
| Right-Lateral-Ventricle | 0.28 | 0.000 | 0.65 | 0.000 | 0.60 (1.77) | 0.50 (1.42) | 6386 | 0.970 |
| Right-Pallidum | 0.59 | 0.000 | 0.71 | 0.000 | 0.29 (1.51) | 0.02 (1.22) | 7089 | 0.489 |
| Right-Putamen | 0.67 | 0.000 | 0.76 | 0.000 | 0.42 (1.58) | 0.09 (0.99) | 7365 | 0.267 |
| Right-Thalamus-Proper | 0.77 | 0.000 | 0.82 | 0.000 | 1.08 (3.01) | 1.37 (3.11) | 6074 | 0.899 |
| Right-VentralDC | 0.73 | 0.000 | 0.72 | 0.000 | 0.07 (0.94) | -0.06 (0.93) | 7203 | 0.368 |
| lh_bankssts_volume | 0.74 | 0.000 | 0.77 | 0.000 | 0.49 (2.05) | 0.45 (1.87) | 6447 | 0.916 |
| lh_caudalanteriorcingulate_volume | 0.70 | 0.000 | 0.63 | 0.000 | 0.06 (0.84) | 0.06 (1.15) | 6367 | 0.970 |
| lh_caudalmiddlefrontal_volume | 0.69 | 0.000 | 0.58 | 0.000 | 0.21 (1.42) | 0.06 (1.13) | 6688 | 0.766 |
| lh_cuneus_volume | 0.78 | 0.000 | 0.69 | 0.000 | 0.38 (1.62) | 0.32 (1.56) | 6485 | 0.916 |
| lh_entorhinal_volume | 0.64 | 0.000 | 0.56 | 0.000 | 0.05 (1.37) | 0.19 (1.63) | 5695 | 0.641 |
| lh_frontalpole_volume | 0.59 | 0.000 | 0.69 | 0.000 | 0.42 (2.68) | 0.70 (2.83) | 5851 | 0.742 |
| lh_fusiform_volume | 0.78 | 0.000 | 0.69 | 0.000 | 0.12 (1.29) | -0.09 (1.05) | 6967 | 0.633 |
| lh_inferiorparietal_volume | 0.79 | 0.000 | 0.80 | 0.000 | 0.20 (1.99) | 0.23 (1.67) | 6467 | 0.916 |
| lh_inferiortemporal_volume | 0.59 | 0.000 | 0.74 | 0.000 | 0.06 (1.22) | 0.04 (1.33) | 6335 | 0.998 |
| lh_insula_volume | 0.66 | 0.000 | 0.62 | 0.000 | 0.17 (1.29) | 0.27 (1.49) | 5695 | 0.641 |
| lh_isthmuscingulate_volume | 0.73 | 0.000 | 0.64 | 0.000 | 0.33 (1.46) | -0.07 (1.22) | 7215 | 0.368 |
| lh_lateraloccipital_volume | 0.74 | 0.000 | 0.49 | 0.000 | 0.15 (1.16) | -0.01 (1.10) | 6766 | 0.742 |
| lh_lateralorbitofrontal_volume | 0.69 | 0.000 | 0.66 | 0.000 | 0.75 (1.95) | 0.52 (1.78) | 6792 | 0.742 |
| lh_lingual_volume | 0.76 | 0.000 | 0.80 | 0.000 | 0.38 (2.09) | 0.53 (1.90) | 5880 | 0.742 |
| lh_medialorbitofrontal_volume | 0.62 | 0.000 | 0.63 | 0.000 | 0.26 (2.18) | 0.28 (1.70) | 6725 | 0.750 |
| lh_middletemporal_volume | 0.66 | 0.000 | 0.64 | 0.000 | 0.25 (1.82) | 0.12 (1.20) | 6590 | 0.872 |
| lh_paracentral_volume | 0.76 | 0.000 | 0.67 | 0.000 | 0.08 (1.14) | -0.01 (1.09) | 6374 | 0.970 |
| lh_parahippocampal_volume | 0.67 | 0.000 | 0.44 | 0.000 | -0.00 (0.89) | -0.12 (0.86) | 6599 | 0.859 |
| lh_parsopercularis_volume | 0.61 | 0.000 | 0.62 | 0.000 | 0.10 (1.45) | 0.01 (1.19) | 6756 | 0.742 |
| lh_parsorbitalis_volume | 0.57 | 0.000 | 0.72 | 0.000 | 0.25 (1.49) | 0.10 (1.35) | 6755 | 0.742 |
| lh_parstriangularis_volume | 0.74 | 0.000 | 0.68 | 0.000 | 0.14 (1.63) | 0.30 (1.38) | 6155 | 0.916 |
| lh_pericalcarine_volume | 0.88 | 0.000 | 0.81 | 0.000 | 1.87 (4.08) | 1.20 (3.41) | 6453 | 0.916 |
| lh_postcentral_volume | 0.76 | 0.000 | 0.72 | 0.000 | 0.10 (0.99) | 0.20 (1.37) | 5708 | 0.662 |
| lh_posteriorcingulate_volume | 0.74 | 0.000 | 0.60 | 0.000 | 0.05 (1.34) | 0.10 (1.00) | 6450 | 0.916 |
| lh_precentral_volume | 0.77 | 0.000 | 0.70 | 0.000 | 0.68 (2.42) | 0.68 (2.17) | 6631 | 0.840 |
| lh_precuneus_volume | 0.53 | 0.000 | 0.63 | 0.000 | -0.08 (1.10) | -0.03 (0.69) | 6520 | 0.916 |
| lh_rostralanteriorcingulate_volume | 0.75 | 0.000 | 0.68 | 0.000 | 0.18 (1.85) | 0.01 (1.14) | 6894 | 0.700 |
| lh_rostralmiddlefrontal_volume | 0.66 | 0.000 | 0.68 | 0.000 | 0.84 (3.51) | 0.73 (3.13) | 6316 | 0.998 |
| lh_superiorfrontal_volume | 0.75 | 0.000 | 0.63 | 0.000 | 0.04 (1.90) | 0.37 (1.32) | 6073 | 0.899 |
| lh_superiorparietal_volume | 0.78 | 0.000 | 0.65 | 0.000 | 0.23 (1.27) | -0.05 (1.08) | 7223 | 0.368 |
| lh_superiortemporal_volume | 0.72 | 0.000 | 0.68 | 0.000 | 0.32 (1.87) | 0.11 (1.06) | 6989 | 0.607 |
| lh_supramarginal_volume | 0.76 | 0.000 | 0.70 | 0.000 | 0.39 (1.65) | 0.40 (1.87) | 6011 | 0.847 |
| lh_temporalpole_volume | 0.56 | 0.000 | 0.71 | 0.000 | -0.16 (0.74) | -0.14 (0.74) | 5950 | 0.766 |
| lh_transversetemporal_volume | 0.60 | 0.000 | 0.83 | 0.000 | 0.18 (1.17) | 0.35 (1.26) | 5909 | 0.750 |
| rh_bankssts_volume | 0.63 | 0.000 | 0.68 | 0.000 | 0.04 (1.05) | 0.12 (0.99) | 6018 | 0.850 |
| rh_caudalanteriorcingulate_volume | 0.62 | 0.000 | 0.73 | 0.000 | 0.01 (0.96) | 0.01 (1.02) | 6443 | 0.917 |
| rh_caudalmiddlefrontal_volume | 0.73 | 0.000 | 0.80 | 0.000 | 0.16 (1.43) | -0.02 (1.23) | 6892 | 0.700 |
| rh_cuneus_volume | 0.48 | 0.000 | 0.73 | 0.000 | -0.00 (1.13) | 0.06 (0.77) | 6196 | 0.919 |
| rh_entorhinal_volume | 0.50 | 0.000 | 0.74 | 0.000 | 0.04 (1.21) | -0.03 (1.16) | 6806 | 0.742 |
| rh_frontalpole_volume | 0.65 | 0.000 | 0.76 | 0.000 | 0.18 (1.76) | 0.09 (1.15) | 6817 | 0.733 |
| rh_fusiform_volume | 0.73 | 0.000 | 0.65 | 0.000 | 0.07 (1.44) | 0.05 (0.84) | 6969 | 0.633 |
| rh_inferiorparietal_volume | 0.75 | 0.000 | 0.55 | 0.000 | -0.03 (0.84) | -0.11 (0.93) | 6269 | 0.970 |
| rh_inferiortemporal_volume | 0.69 | 0.000 | 0.68 | 0.000 | 0.09 (1.17) | 0.02 (1.19) | 6324 | 0.998 |
| rh_insula_volume | 0.74 | 0.000 | 0.80 | 0.000 | 0.11 (1.33) | 0.22 (1.49) | 5901 | 0.750 |
| rh_isthmuscingulate_volume | 0.61 | 0.000 | 0.68 | 0.000 | 0.11 (1.29) | 0.12 (1.13) | 6493 | 0.916 |
| rh_lateraloccipital_volume | 0.80 | 0.000 | 0.77 | 0.000 | 0.39 (1.57) | 0.18 (1.33) | 7237 | 0.368 |
| rh_lateralorbitofrontal_volume | 0.77 | 0.000 | 0.76 | 0.000 | 1.20 (2.91) | 0.31 (2.58) | 7030 | 0.548 |
| rh_lingual_volume | 0.79 | 0.000 | 0.72 | 0.000 | 0.10 (1.23) | -0.05 (0.76) | 7177 | 0.374 |
| rh_medialorbitofrontal_volume | 0.57 | 0.000 | 0.84 | 0.000 | 0.25 (1.63) | 0.39 (1.46) | 6085 | 0.909 |
| rh_middletemporal_volume | 0.62 | 0.000 | 0.75 | 0.000 | 0.11 (1.44) | 0.22 (1.34) | 6500 | 0.916 |
| rh_paracentral_volume | 0.76 | 0.000 | 0.56 | 0.000 | -0.00 (0.74) | -0.07 (1.04) | 6161 | 0.916 |
| rh_parahippocampal_volume | 0.68 | 0.000 | 0.76 | 0.000 | -0.04 (0.93) | -0.04 (0.99) | 6519 | 0.916 |
| rh_parsopercularis_volume | 0.62 | 0.000 | 0.69 | 0.000 | 0.15 (1.38) | 0.06 (1.45) | 6698 | 0.766 |
| rh_parsorbitalis_volume | 0.59 | 0.000 | 0.72 | 0.000 | 0.08 (1.30) | 0.18 (1.93) | 6063 | 0.889 |
| rh_parstriangularis_volume | 0.56 | 0.000 | 0.70 | 0.000 | 0.27 (1.69) | 0.33 (1.39) | 6324 | 0.998 |
| rh_pericalcarine_volume | 0.87 | 0.000 | 0.83 | 0.000 | 1.04 (3.13) | 0.77 (3.88) | 6103 | 0.916 |
| rh_postcentral_volume | 0.73 | 0.000 | 0.72 | 0.000 | -0.05 (1.01) | 0.01 (1.13) | 6302 | 0.998 |
| rh_posteriorcingulate_volume | 0.56 | 0.000 | 0.70 | 0.000 | 0.01 (1.00) | -0.00 (1.34) | 6242 | 0.963 |
| rh_precentral_volume | 0.74 | 0.000 | 0.84 | 0.000 | 0.66 (2.66) | 0.81 (2.39) | 6264 | 0.970 |
| rh_precuneus_volume | 0.71 | 0.000 | 0.71 | 0.000 | 0.13 (1.00) | -0.02 (0.95) | 6942 | 0.641 |
| rh_rostralanteriorcingulate_volume | 0.71 | 0.000 | 0.81 | 0.000 | 0.00 (1.03) | -0.00 (1.20) | 6185 | 0.916 |
| rh_rostralmiddlefrontal_volume | 0.64 | 0.000 | 0.64 | 0.000 | 0.43 (2.50) | 0.46 (1.46) | 6759 | 0.742 |
| rh_superiorfrontal_volume | 0.79 | 0.000 | 0.65 | 0.000 | 0.30 (2.03) | 0.37 (2.07) | 6109 | 0.916 |
| rh_superiorparietal_volume | 0.72 | 0.000 | 0.72 | 0.000 | 0.01 (1.13) | 0.10 (0.80) | 6485 | 0.916 |
| rh_superiortemporal_volume | 0.61 | 0.000 | 0.68 | 0.000 | 0.18 (1.32) | 0.05 (1.31) | 6471 | 0.916 |
| rh_supramarginal_volume | 0.74 | 0.000 | 0.71 | 0.000 | 0.16 (1.32) | 0.01 (0.85) | 7240 | 0.368 |
| rh_temporalpole_volume | 0.74 | 0.000 | 0.62 | 0.000 | 0.13 (1.81) | 0.13 (0.88) | 6819 | 0.733 |
| rh_transversetemporal_volume | 0.79 | 0.000 | 0.79 | 0.000 | 0.39 (1.74) | 0.38 (1.78) | 6397 | 0.963 |
| 3rd-Ventricle | 0.38 | 0.000 | 0.93 | 0.000 | 0.73 (1.59) | 0.97 (1.88) | 5572 | 0.511 |
| 4th-Ventricle | 0.29 | 0.000 | 0.70 | 0.000 | 0.01 (0.95) | 0.24 (1.07) | 5924 | 0.762 |
| CC_Anterior | 0.75 | 0.000 | 0.75 | 0.000 | 0.04 (0.97) | 0.07 (1.16) | 6014 | 0.847 |
| CC_Central | 0.79 | 0.000 | 0.82 | 0.000 | 0.68 (2.36) | 0.80 (2.66) | 5760 | 0.700 |

SW: Shapiro Wilk’s test; HC: healthy controls; Med: median; interquartile range; Mann-U; Mann-Whitney U test; Adj: adjusted.

# Table S9. Shapiro’s Wilk test for positive, negative and dementia symptoms.

| Symptom | Test Statistic | p-value |
| --- | --- | --- |
| Positive | 0.78 | <0.001 |
| Negative | 0.77 | <0.001 |
| MMSCORE | 0.89 | <0.001 |

# Table S10. Correlation between statistically significant ROI between Alzheimer’s disease and healthy controls and MMSE.

| ROI | r | p value |
| --- | --- | --- |
| Left-Hippocampus | -0.28 | **0.042** |
| Left-Inf-Lat-Vent | -0.13 | 0.391 |
| Right-Amygdala | -0.15 | 0.293 |
| Right-Hippocampus | -0.01 | 0.962 |
| Right-Inf-Lat-Vent | -0.14 | 0.341 |
| lh_inferiorparietal_volume | -0.17 | 0.241 |
| rh_inferiortemporal_volume | -0.15 | 0.297 |
| Left-Amygdala | -0.28 | **0.045** |
| lh_inferiortemporal_volume | -0.34 | **0.017** |

# Table S11. Correlation between statistically significant ROI between Schizophrenia and healthy controls and positive/ negative symptoms.

| ROI | Positive symptoms | | Negative symptoms | |
| --- | --- | --- | --- | --- |
|  | r | p value | r | p value |
| Right-Hippocampus | 0.22 | **0.034** | 0.20 | **0.048** |
| Left-Hippocampus | 0.15 | 0.136 | 0.05 | 0.647 |
| Left-Putamen | -0.01 | 0.936 | 0.01 | 0.998 |

# Table S12. Mean cosine similarity of deviation scores amongst patients and controls.

|  | Alzheimer's disease | | Schizophrenia | |
| --- | --- | --- | --- | --- |
|  | Healthy Controls | Patients | Healthy Controls | Patients |
| All ROIs | 0.31 (0.25) | 0.54 (0.22) | 0.40 (0.17) | 0.32 (0.15) |
| Ventricles | 0.56 (0.24) | 0.72 (0.20) | 0.62 (0.23) | 0.55 (0.22) |
| Cerebellum | 0.67 (0.22) | 0.71 (0.20) | 0.67 (0.23) | 0.71 (0.21) |
| Corpus callosum | 0.59 (0.24) | 0.60 (0.24) | 0.60 (0.25) | 0.61 (0.23) |
| Subcortical regions | 0.42 (0.20) | 0.60 (0.20) | 0.47 (0.21) | 0.38 (0.20) |
| Insula | 0.79 (0.23) | 0.83 (0.18) | 0.81 (0.21) | 0.81 (0.22) |
| Frontal lobe | 0.41 (0.16) | 0.39 (0.16) | 0.42 (0.16) | 0.40 (0.17) |
| Parietal lobe | 0.51 (0.20) | 0.53 (0.19) | 0.51 (0.20) | 0.49 (0.21) |
| Temporal lobe | 0.42 (0.18) | 0.43 (0.19) | 0.42 (0.17) | 0.42 (0.17) |
| Occipital lobe | 0.55 (0.21) | 0.53 (0.21) | 0.59 (0.22) | 0.56 (0.23) |
| Cingulate cortex | 0.53 (0.21) | 0.56 (0.22) | 0.52 (0.21) | 0.54 (0.21) |
